# Comparative genomics and transcriptomics of *Pichia pastoris*

**DOI:** 10.1186/s12864-016-2876-y

**Published:** 2016-08-05

**Authors:** Kerry R. Love, Kartik A. Shah, Charles A. Whittaker, Jie Wu, M. Catherine Bartlett, Duanduan Ma, Rachel L. Leeson, Margaret Priest, Jonathan Borowsky, Sarah K. Young, J. Christopher Love

**Affiliations:** 1 Koch Institute for Integrative Cancer Research, Massachusetts Institute of Technology, 76-253, 77 Massachusetts Avenue, Cambridge, MA 02139 USA; 2 The Barbara K. Ostrom (1978) Bioinformatics and Computing Facility in the Swanson Biotechnology Center, Massachusetts Institute of Technology, Cambridge, MA 02139 USA; 3 The Broad Institute of MIT and Harvard, Cambridge, MA 02142 USA

**Keywords:** RNA-Seq, Transcriptome, *Pichia pastoris*, *Komagataella pastoris*, *Komagataella phaffii*, Self-Organizing Maps (SOMs), Cultivation dependent expression, Secretome, Gene Set Enrichment Analysis (GSEA)

## Abstract

**Background:**

*Pichia pastoris* has emerged as an important alternative host for producing recombinant biopharmaceuticals, owing to its high cultivation density, low host cell protein burden, and the development of strains with humanized glycosylation. Despite its demonstrated utility, relatively little strain engineering has been performed to improve *Pichia*, due in part to the limited number and inconsistent frameworks of reported genomes and transcriptomes. Furthermore, the co-mingling of genomic, transcriptomic and fermentation data collected about *Komagataella pastoris* and *Komagataella phaffii*, the two strains co-branded as Pichia, has generated confusion about host performance for these genetically distinct species. Generation of comparative high-quality genomes and transcriptomes will enable meaningful comparisons between the organisms, and potentially inform distinct biotechnological utilies for each species.

**Results:**

Here, we present a comprehensive and standardized comparative analysis of the genomic features of the three most commonly used strains comprising the tradename *Pichia: K. pastoris* wild-type*, K. phaffii* wild-type, and *K. phaffii* GS115. We used a combination of long-read (PacBio) and short-read (Illumina) sequencing technologies to achieve over 1000X coverage of each genome. Construction of individual genomes was then performed using as few as seven individual contigs to create gap-free assemblies. We found substantial syntenic rearrangements between the species and characterized a linear plasmid present in *K. phaffii*. Comparative analyses between *K. phaffii* genomes enabled the characterization of the mutational landscape of the GS115 strain. We identified and examined 35 non-synonomous coding mutations present in GS115, many of which are likely to impact strain performance. Additionally, we investigated transcriptomic profiles of gene expression for both species during cultivation on various carbon sources. We observed that the most highly transcribed genes in both organisms were consistently highly expressed in all three carbon sources examined. We also observed selective expression of certain genes in each carbon source, including many sequences not previously reported as promoters for expression of heterologous proteins in yeasts.

**Conclusions:**

Our studies establish a foundation for understanding critical relationships between genome structure, cultivation conditions and gene expression. The resources we report here will inform and facilitate rational, organism-wide strain engineering for improved utility as a host for protein production.

**Electronic supplementary material:**

The online version of this article (doi:10.1186/s12864-016-2876-y) contains supplementary material, which is available to authorized users.

## Background

Societal pressures to lower healthcare costs, enable precision medicine, and foster economic growth in emerging markets, combined with the projected market demands for both new biopharmaceutical drugs (cardiovascular and neurodegenerative diseases) and biosimilars, motivate continued innovation in manufacturing of biopharmaceutical drugs [1, 2]. Engineering alternative hosts other than conventional mammalian systems such as Chinese hamster ovary (CHO) cells could facilitate new streamlined processes that allow for fast production of high-quality proteins with simplified operations and reduced costs [3, 4]. *Pichia pastoris* is a promising eukaryotic host used today to produce marketed products throughout the world [5, 6], including FDA-approved Jetrea® and Kalbitor®. Despite its commercial successes to date [7], advanced engineering of its secretory capacity, metabolic health, and pathways for post-translational modifications of proteins are still needed to realize its potential as a routine alternative to CHO cells, particularly for proteins with increased complexity [8].

A critical issue impeding efforts to further understand the biology of this yeast, and engineer its metabolic state and secretion system, is the entwinement of learning related to two distinct organisms (*Komagataella phaffii* and *K. pastoris*) [9, 10]. Previously defined, and now co-branded, as *Pichia pastoris*, both species are used for heterologous protein expression; understanding of their behaviors in expression and fermentation are assumed to apply to each. The GS115 strain—an auxotrophic mutant of *K. phaffii* (NRRL Y-11430) derived by chemical mutagenesis—is also widely used for protein production and further complicates the literature [11].

Previous characterization of the genomes and transcriptomes for these three strains have established independent tools for working with each. Pyrosequencing of *K. phaffii* GS115 provided the first assembled genome with annotated genes based on *Sacchromyces cerevisiae* [12]. Short-read sequencing of a *K. pastoris* strain (DSMZ 70382) yielded super contigs without a genome-level assembly [13]. A similar approach for wildtype K. phaffii (NRRL Y-11430; CBS7435) refined the assembly of GS115, and included a fully annotated mitochondrial genome and methanol utilization pathway [14]. Recent studies have identified other potential functional elements within the genome, including autonomously replicating sequences (ARS) in a ura3-deficient mutant GS115 (JC308) [15], as well as two IRES elements [16]. While *Pichia*-specific microarrays have been reported [17, 18], transcriptional analyses have relied primarily on microarrays based on *S. cerevisiae* [19, 20], and there is limited published knowledge on how gene expression of null strains compare during growth on relevant carbon sources, namely glycerol, glucose, and methanol. Despite the range of studies on specific strains and fermentation conditions, including two reports using data generated by RNA-seq [16, 21], unified datasets of genomic features and transcriptional landscapes are scarce for the two organisms [20]. Without a common genomic and transcriptional framework, biological engineering of these strains to enhance their specific productivity and metabolic state remains difficult.

Based on these considerations, we present here a comprehensive and standardized comparative analysis of the genomic and transcriptomic features of the parental strains of *K. phaffii* and *K. pastoris*, as well as a detailed map of the mutational landscape of GS115 relative to its parental strain, wildtype *K. phaffii*. This resource provides a standardized and cohesive foundation for future strain engineering to help overcome secretory capacity limitations and improve metabolic pathways for desirable growth and quality-by-design (QbD) production.

## Results and discussion

### Genome and transcriptome sequencing, assembly, and annotation

We sequenced the genomes of *K. phaffii* (wildtype: NRRL Y-11430 or ATCC 76273 and GS115: ATCC 20864) and *K. pastoris* (wildtype: NRRL Y-1603 or ATCC 28485) using a combination of long-read (PacBio) and short-read (Illumina) sequencing technologies (Additional file 1: Table S1). For all three strains, poly(A)-enriched, strand-specific cDNA was also sequenced (RNA-Seq) from triplicate batch cultivations in various carbon sources (Additional file 2: Figure S1; Additional file 3: Table S2). Both the genome sequencing and *de novo* assembled transcript models from the initial outgrowth were used for the assembly and initial annotation of each genome (Table 1) [22]. For each genome, the PacBio sequencing provided more than 100x coverage and the Illumina as high as 1,800x coverage; more than 78 % of reads aligned in *post hoc* validation. The exceptional coverage and long reads yielded a maximum of 11 total contigs from which to assemble the genomes—more than ten times fewer than previous reported assemblies. The genome of *K. pastoris* is 9.6 Mbp in size, slightly larger than that of *K. phaffii* (9.4 Mbp), consistent with previous reports [23]. There were no gaps in coverage remaining in the four major chromosomes for each species, though there were 4–5 small contigs for each strain containing rDNA or telomeric sequences that we were unable to assign to any major chromosome due to their highly repetitive content despite manual curation of the assembly using long reads from PacBio sequencing.

**Table 1:** Genome assembly and annotation statistics for major chromosomes

	*K. Pastoris*	*K. Phaffii*	
		WT	GS115
Genome Size (Mb)	9.6	9.4	9.4
Chromosomes	4	4	4
Contigs	11	7	9
Pacbio Coverage	168x	118x	207x
Illumina Coverage	312x	1869x	1498x
Coding (%)	78.6	79.9	79.5
Coding Genes	5241	5167	5183
tRNA Genes	122	123	123
5S rRNA Genes	23	21	21
GC%	41.5 %	41.3 %	41.3 %

The annotations of each species yielded 5,241 genes in *K. pastoris* and 5,167 in *K. phaffii*. These were linked to existing publicly-available annotated genomes using BLAST (Additional file 4: Table S3). Sequence clustering at the mRNA level was used to compare the two species and the GS115 mutant to identify orthologs between strains. Using this approach there were 4,601 orthologs at the gene level (1:1:1 association between strains), with 4,996 orthologs (1:1) between *K. phaffii* and the mutant GS115. Manual analysis of the clustering resulted in further annotation of 48 orthologs between *K. pastoris* and *K. phaffii.* The remaining gene differences between these strains may be attributed to artifacts incurred during annotation of adjacent genes, including fragmented gene prediction or incomplete UTR annotation. (For a detailed discussion of manual orthology assignment, see Orthology Assignment and Gene Naming in Methods.) Seven genes were found only in the *K. phaffii* wildtype and are attributed to a linear plasmid in this strain (see further discussion below). Additionally, one gene in *K. phaffii* wildtype is likely inactivated in the GS115 mutant due to a frame shift mutation. Three hundred ninety-eight genes appear to be species-specific, occurring only in either *K. pastoris* or *K. phaffii*, and are not a result of data contamination [24]. Our constructed phylogeny confirmed that *K. pastoris* and *K. phaffii* are closely related, but distinct species (Additional file 2: Figure S2).

Of the orthologs between the species, 3,556 genes were named by association to *S. cerevisiae*. An additional 30 genes, associated either with flocculation [25], or central carbon metabolism [14, 26] (including the methanol utilization (MUT) pathway) were manually assigned, though these genes may not correspond to *S. cerevisiae* (see Additional file 5: Table S4 for a complete list of named orthologs). Alignment to the annotated genomes of next-nearest neighbor (e.g., *Kluyveromyces lactis* or *Hansenula polymorpha*) could improve the curation. Using an 80 % identity cut-off to establish ~4600 1:1 orthologs, there was a reasonable conservation at the nucleotide level between the two species (~91 % average base pair identity; Additional file 2: Figure S3). The alpha factor protein (Chr 2 both species) is <85 % identical at both the nucleotide and amino acid level. This relatively low identity results from two repeated sequence motifs present in *K. phaffii*, but not in *K. pastoris*—a feature common in other proteins identified in *K. pastoris* as well (e.g., flocculation genes). Two commonly used promoters are highly conserved between the species, but not identical (P_AOX1_, Chr 4, 90 % identity, and P_GAPDH_, Chr 2, 88 % identity). The observed variances between species imply that precise sequences of genes and loci are important for engineering specific sites in each species.

### Key features of *Komagataella* genomes and transcriptomes

#### Genome characteristics and rearrangements between species

**Fig. 1:**
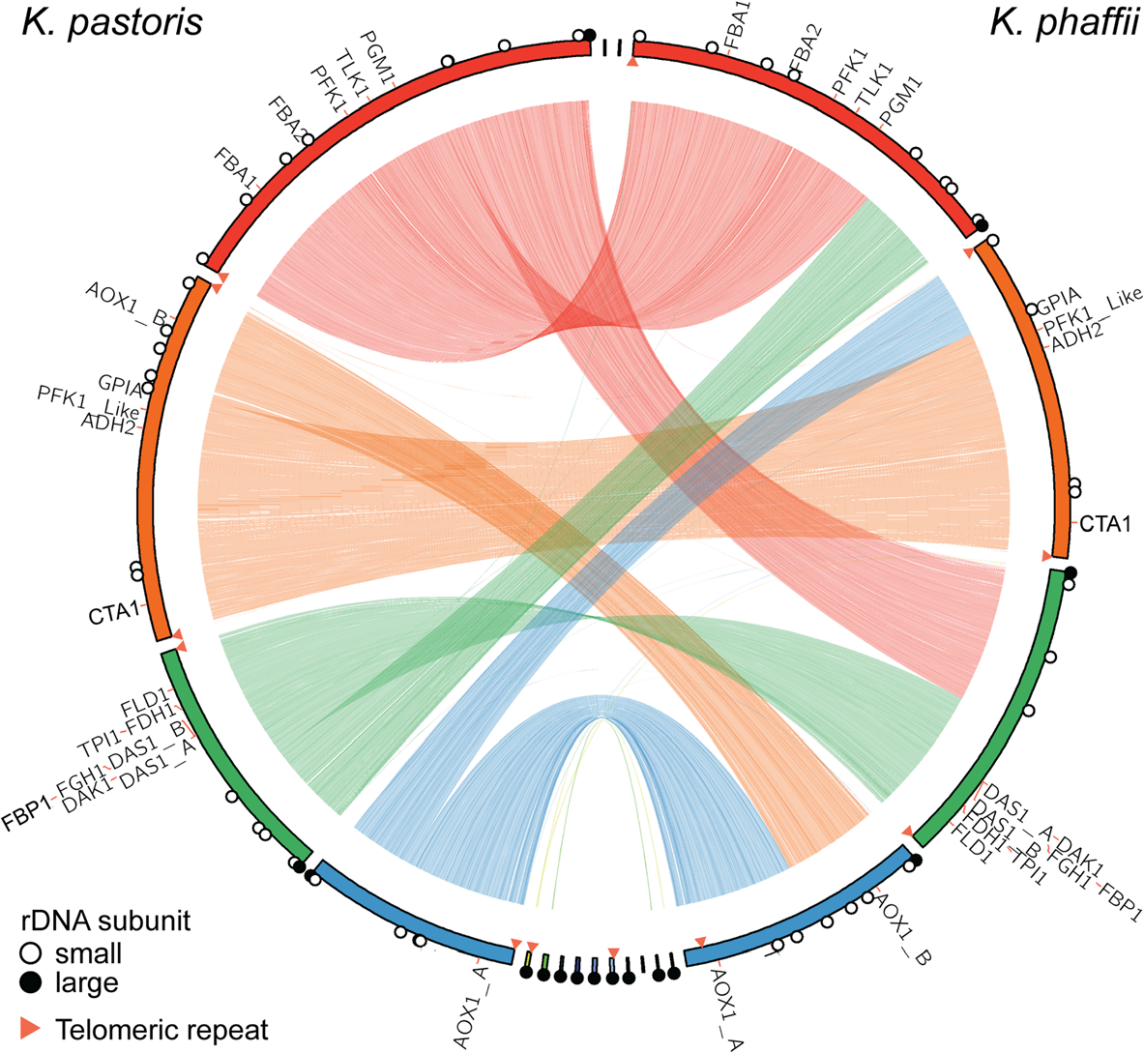
Comparative genome structure of *K. pastoris* and *K. phaffii*. Circos plot indicating the sequence alignment between *K. pastoris* and *K. phaffii* marked with methanol utilization pathway (MUT) genes. Functional genetic elements marked on the plots include: small rDNA subunits (*white circles*), large rDNA subunits (*black circles*), and telomeric repeats (*orange triangles*)

We then compared the assembled genomes of *K. pastoris* and *K. phaffii* and found substantial syntenic rearrangements between the two species (Fig. 1). The breakpoints of these rearrangements appear to lie adjacent to 5S rDNA loci. The internal structure of the chromosomes, however, were largely conserved. As an example, the MUT pathway retains its gene order and relative orientation within the chromosome despite its relocation within the genome. Examples of gross chromosomal rearrangements (GCRs) caused by unstable repetitive loci under conditions of environmental stress have been reported in the brewing industry [27]. There were no gene copy number variations among the three strains (Additional file 2: Figure S4). Both species have extremely similar codon usage with 122–123 tRNA genes identified (Additional file 2: Figure S5); the usage for *K. phaffii* agreed with previous reports [12].

#### Identification of functional DNA elements

We identified 100–340 bp of telomeric repeats (TGGATGC) on chromosomal termini of all three strains. These homogenous repeated sequences are similar to ones found in closely related yeasts *Yarrowia lipolytica* (TTAGTCAGGG) and *H. polymorpha* (TGGCGGGG), and unlike the heterogeneous telomeric repeat sequences found in *S. cerevisiae* ([TG]_2-3_[TG]_1-6_) and *Schizosaccharomyces pombe* (TTAC[A][C]G_2-8_) [28]. An rDNA cluster (containing 18S, 5.8S and 16S rRNA genes) was located at a subtelomeric position on Chr 1, 3 and 4 in *K. pastoris*, on Chr 1, 3, and 4 in wildtype *K. phaffii* and on Chr 1 in the *K. phaffii* GS115 strain. We also located 21–23 copies of the 5S rRNA gene dispersed throughout the genome.

#### Characterization of a linear plasmid in *K. phaffii* strains

**Fig. 2:**
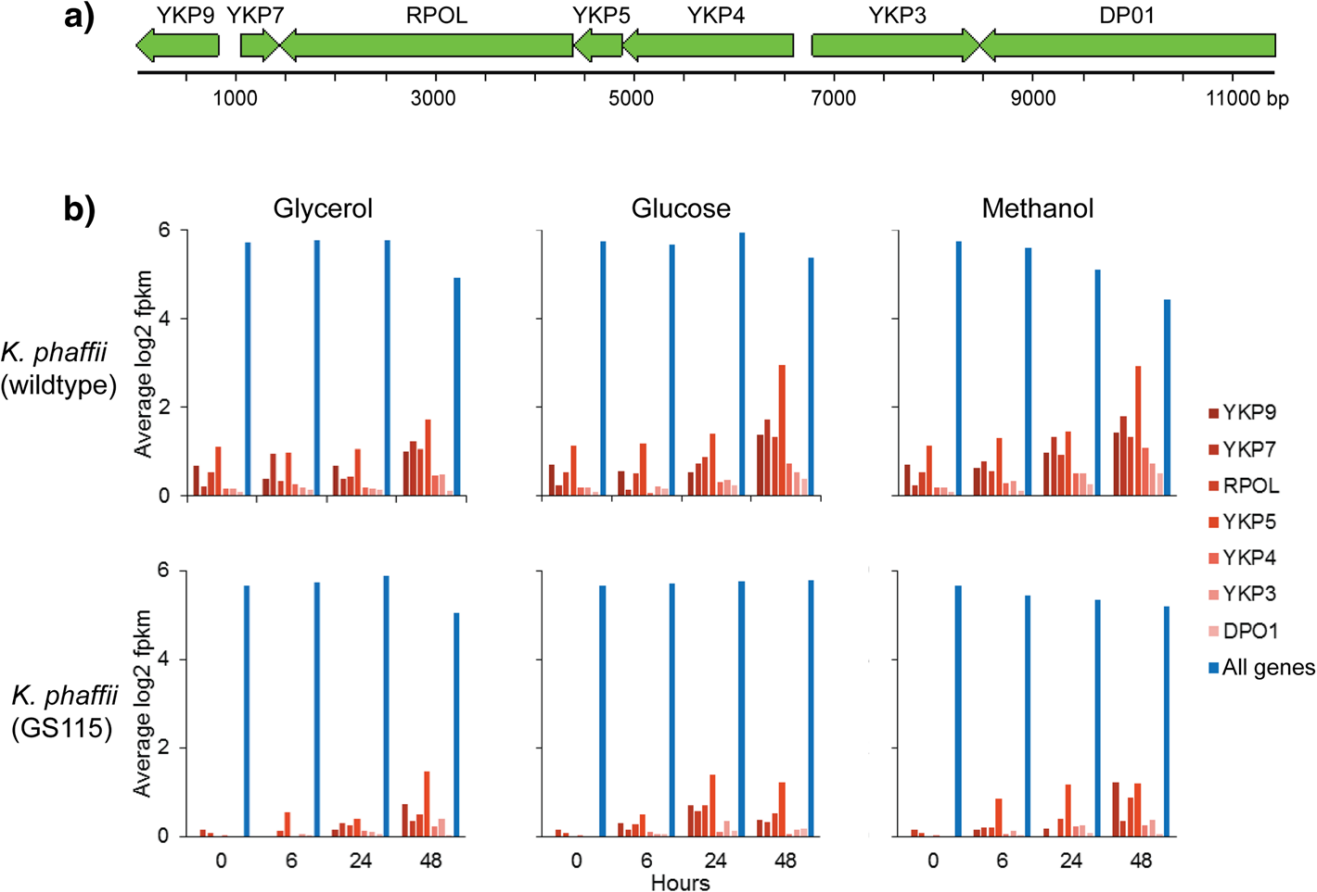
Linear plasmid annotation and expression in *K. phaffii*. **a) ** Schematic representation of the 11 kb linear plasmid annotated with seven genes homologous to the *K. lactis* killer plasmid. **b) ** Comparison of gene expression between genes located on the killer plasmid in i) wild type *K. phaffii* or ii) *K. phaffii* GS115 and the average gene expression among chromosomally-located genes in each species during cultivation on three different carbon sources

We found a highly AT-rich (~72 %) contig (~11 Kb) in the wildtype *K. phaffii* assembly devoid of both rDNA and telomeric sequences that did not align to other chromosomal sequences in any of the three genomes. While naturally-occurring episomal plasmids have not previously been used in *Komagataella* strains, an undescribed 20 kb linear plasmid was recently reported for *K. phaffii* [14]. Our annotation pipeline predicted the presence of seven genes within this contig (Fig. 2a), all of them homologous to genes of the well-known linear dsDNA killer plasmid system from *K. lactis* [29]. Five out of these seven genes have no known function, but two code for putative subunits of DNA and RNA polymerase, respectively. The high AT content throughout the contig, particularly at its termini, suggests that this linear plasmid may have a distinct mechanism of replication and self-maintenance. RNA-seq revealed that all seven genes express at extremely low levels compared to average genome-wide expression (Fig. 2b). Gene expression increased modestly during batch cultivation, but did not vary substantially with carbon source. It is currently unclear if any of these genes encode a secreted killer toxin or if the presence of this plasmid confers either killer activity or a selective advantage to the *K. phaffii* host strain. The low expression means it is not likely useful for heterologous protein expression, but could provide sites for introducing genome editing tools.

Although the linear plasmid was not assembled in the GS115 genome, we found transcripts encoding GS115 orthologs of linear plasmid genes. To investigate the relative stability and quality of these sequences in the two strains, we calculated the rate of mismatches detected in the Illumina reads from each strain. Despite similar mismatch rates in the plasmid genes for both strains, there were many fewer aligned reads detected for the plasmid in GS115 on both sequencing platforms (Additional file 6: Table S5). This result suggests a lower copy number present for this plasmid in GS115. RNA-seq data appeared to corroborate this hypothesis; expression levels for the majority of the seven genes were substantially lower in GS115 (Fig. 2b). There was no evidence for the plasmid in *K. pastoris*: No PacBio reads aligned to the plasmid, no transcripts were detected for any of the seven genes encoded on the plasmid, and there were no positive BLAST hits.

#### Identification of alternatively spliced genes

Based on detection of gapped alignment in our initial annotation, we found that only 21 genes from either *K. pastoris* or *K. phaffii* exhibited splice variants that altered coding sequences (CDS), with five of these genes having alternative isoforms present in both species. We did not observe any significant changes in isoform dominance of these alternatively spliced genes attributable to our batch cultivation from either carbon source or duration (Additional file 2: Figure S6). There was no functional enrichment observed among these alternatively spliced genes based on assignment of GO terms. An additional 175 potential CDS-altering variants were detected by stringent manual review of the RNA-Seq data (Additional file 7: Table S6), along with different splice junctions for four genes identified in the initial analysis. These putative variants require additional validation to confirm alternative splicing and to determine the exact number of isoforms present for each species.

### Impact of genomic structure on gene expression

**Fig. 3:**
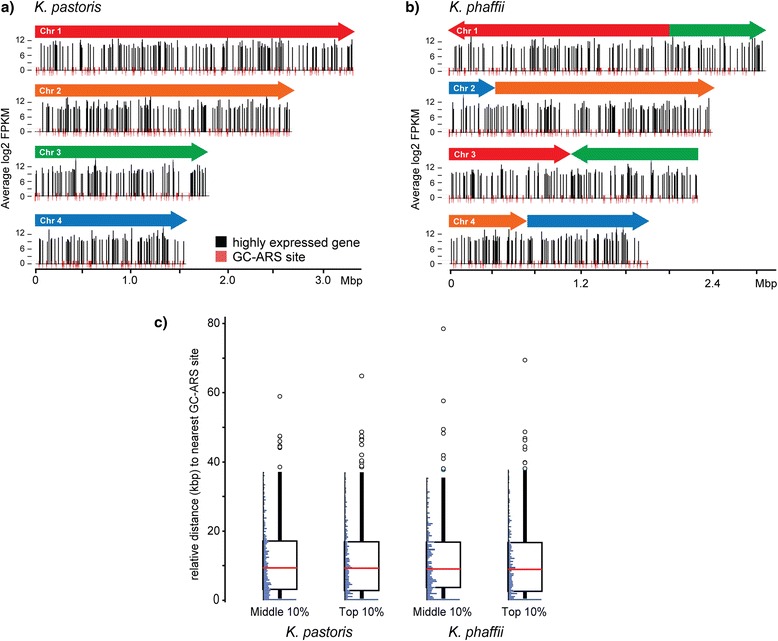
Gene expression as a function of chromosomal location. Map of chromosomal location (base pair identity) for the most highly expressed genes (top 10 % expression) in **a**) *K. pastoris* and **b**) *K. phaffii. Black lines* indicate gene expression level at 24 h time point during batch cultivation in methanol. *Red lines* indicate locations of GC-rich autonomously replicating sequence (GC-ARS) motifs identified by BLAST. **c)** Box and whisker plot of the relative distance to the nearest GC-ARS motif in *K. pastoris and K. phaffii* for genes expressed at average levels genome-wide (45–55 % of max expression) and for the most highly expressed genes (top 10 % expression). Histograms of relative distances to GC-ARS motifs are shown alongside box plots for each gene set analyzed

Since there were substantial differences in the chromosomes between *K. pastoris* and *K. phaffii*, we next investigated if there was any influence of chromosomal organization on transcriptional activity during cultivation. The specific expression of regions within the genomes could guide the selection of loci for inserting heterologous proteins. We initially, therefore, decided to focus our efforts on the most highly expressed genes (top 10 %) from our RNA-seq data collected during fermentation in each carbon source and mapped the locations of these genes to the chromosomes of each species. Neither species showed any global regions of transcriptional activity specific to any carbon source, but rather highly expressed genes distributed across the four chromosomes. (Additional file 2: Figure S7) We also examined the potential influence of specific functional elements within the chromosomes on genome-wide transcriptional activity, namely autonomously replicating sequences (ARS). Recently, GC-rich ARS sites associated with transcription were identified for *Pichia pastoris* [15], but a positive correlation between gene expression and replication has not been established. We mapped the GC-rich ARS motifs to our assembled genomes for both species (Additional file 8: Table S7) by BLASTing the reported consensus sequences. Interestingly, their locations did not correlate with increased gene expression in any carbon source (Fig. 3). This finding suggests that the GC-ARS motif may not impact gene expression directly, but could act via regulation of other nearby functional elements, including transcription factors.

### Impact of cultivation conditions on selective expression

While transcriptional activity did not localize to a specific site or functional element within either genome, we did observe subtle differences in the chromosomal profiles of highly expressed genes during cultivation in different carbon sources (Additional file 2: Figure S7). Transcriptomes are expected to vary with cultivation conditions and the use of different carbon sources provides a means to alter gene expression [30]. Understanding these variances could guide host engineering for improved heterologous protein production, including identification of promoter elements tuned to carbon utilization or metabolic pathways needing enhancement, some aspects of which have already been explored [31–33].

**Fig. 4:**
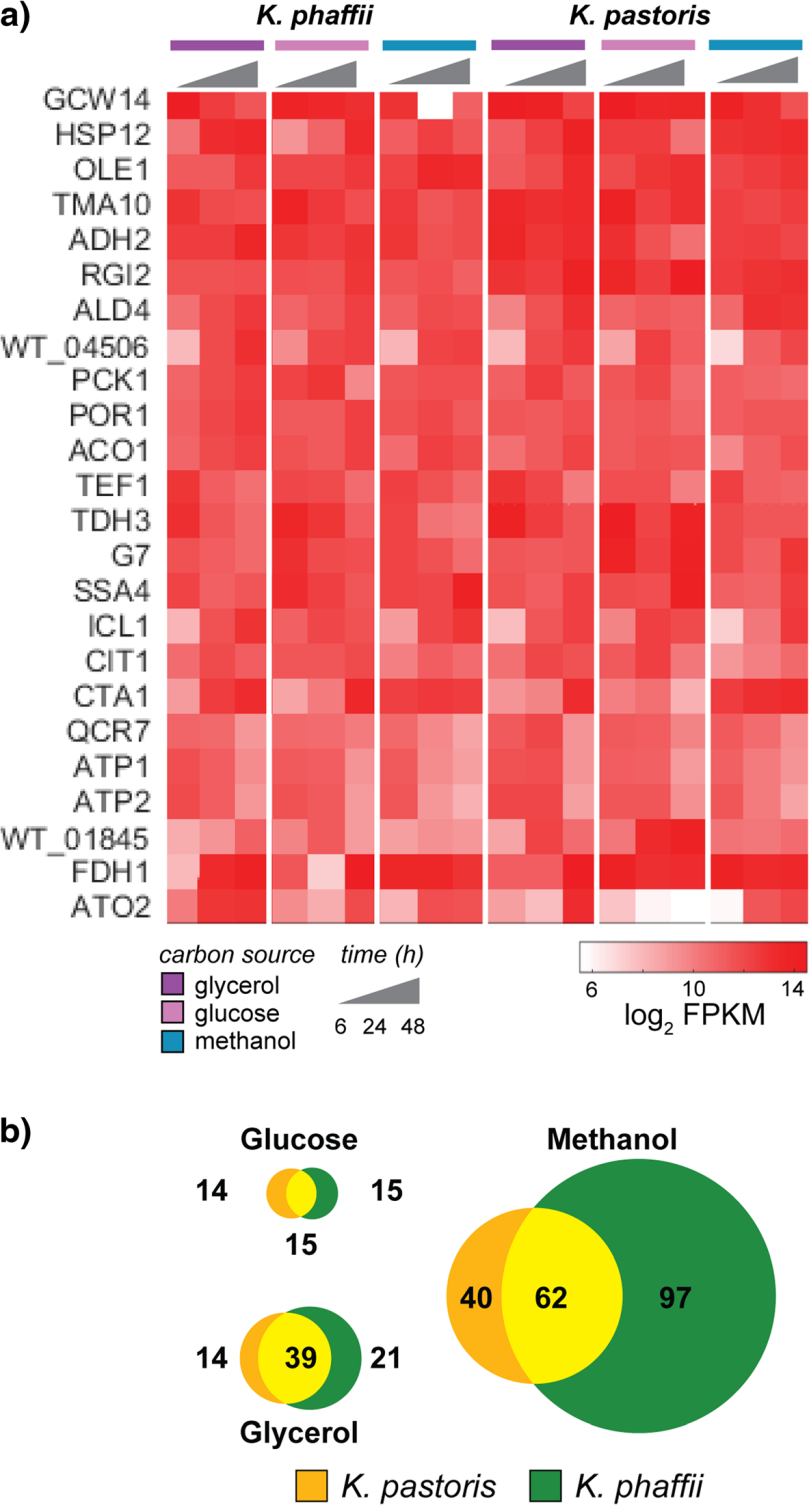
Gene expression in *K. pastoris* and *K. phaffii* as a function of cultivation conditions. **a) ** Heat map of gene expression (log2 fpkm) for the most highly expressed genes at in both strains. Gene expression is shown as a function of batch growth in glycerol, glucose or methanol during a 48 h cultivation period. **b) ** Venn diagrams depicting the intersection between *K. pastoris* (*orange*) and *K. phaffii* (*green*) for genes that are highly (top 10 % expression) and differentially expressed (log2-fold change > 2, *p* < 0.05) during fermentation on a particular carbon source. Circle size is proportional to the total number of genes present for a given condition

We compared gene expression during cultivation for all 1:1 orthologs among each carbon source used, both within a species, and across species cultivated in the same carbon source. The most highly transcribed genes in each organism were consistently highly expressed across all carbon sources; these genes were also consistently expressed over time following transitions between carbon sources (Fig. 4a). Remarkably, only 10 out of these 24 genes have previously been described as useful promoter sequences for protein expression in *Pichia* [5, 32–36]. These genes were associated with GO terms for central metabolism, transport, and stress response, suggesting housekeeping functions. These genes may represent useful promoters for engineering in either species, though disrupting the native loci of each could disrupt essential functions within the cell.

We then sought to identify genes selectively expressed in a particular carbon source (Fig. 4b). Such genes represent promoters (or loci) for use in expression of heterologous proteins during growth in a particular carbon source condition. Methanol has historically been useful as a carbon source to induce heterologous protein expression in *Pichia* [37]. Recently, it was shown that methanol-based cultivation associates with higher levels of translational activity over that observed in glucose- or glycerol-based cultivation [30]. This finding could imply a more extensive transcriptional activation specific to methanol cultivation. Indeed, we confirmed that there were two- to threefold more genes selectively and highly expressed in methanol, depending on the species.

Genes related to transport, lipid metabolism, central metabolism and cellular amino acid metabolic processes were consistently highly expressed in all cultivations, but no categories of GO terms dominated the genes highly expressed in a particular carbon source (Additional file 9: Table S8). During cultivation in methanol, peroxisomal genes (noted similarly in [26]), protein folding and stress response genes were enriched, but also many genes related to diverse cellular activities. Genes specifically enriched during cultivation in glycerol or glucose showed less diversity among GO terms, but still no apparently dominant pathways.

Our analysis revealed all previously reported carbon source-specific promoters used in heterologous protein expression [5, 32–36] in yeasts, but more than 90 % of the genes selectively expressed in any particular carbon source have not been previously used as promoters. Nearly half of these previously unreported genes may lack orthologs in *S. cerevisiae*, and thus, understanding of what pathways are highly active in particular growth conditions will require further studies. Furthermore, we observed key differences in gene expression between the two species. For example, only 62 out of 199 genes selectively expressed in methanol are common among the two strains (Fig. 4b). These differences imply that strain engineering, including promoter selection, must be tuned for each species.

### Biological pathways active during cultivation

To further understand how groups of genes or pathways varied during the batch cultures, we clustered the expression data from each cultivation for each organism using self-organizing maps (SOMs) [38]. During the pre-processing of the expression data for this analysis, we noticed that only 120 genes in *K. pastoris* and 72 in *K. phaffii* (of the 4,600 annotated orthologs between the species) are unexpressed during cultivation (Additional file 2: Figure S8A and S8B). This result implies that these strains are expressing ~98 % of their genome all the time. A minimum and non-degenerate number of clusters (Additional file 2: Figure S8C) was achieved for each organism and cultivation condition to group genes that were changing expression similarly (Additional file 2: Figures S9 and S10). Each of these clusters represents a different expression phenotype within a particular cultivation condition—for example, genes consistently increasing expression over time (Map 8, Glucose; Additional file 2: Figure S10) or genes consistently decreasing expression over time (Map 1, Glucose; Additional file 2: Figure S10). Interestingly, both organisms had a similar number of clusters for each specific carbon source; maps attributed to each carbon source also had similar shapes for both organisms.

**Fig. 5:**
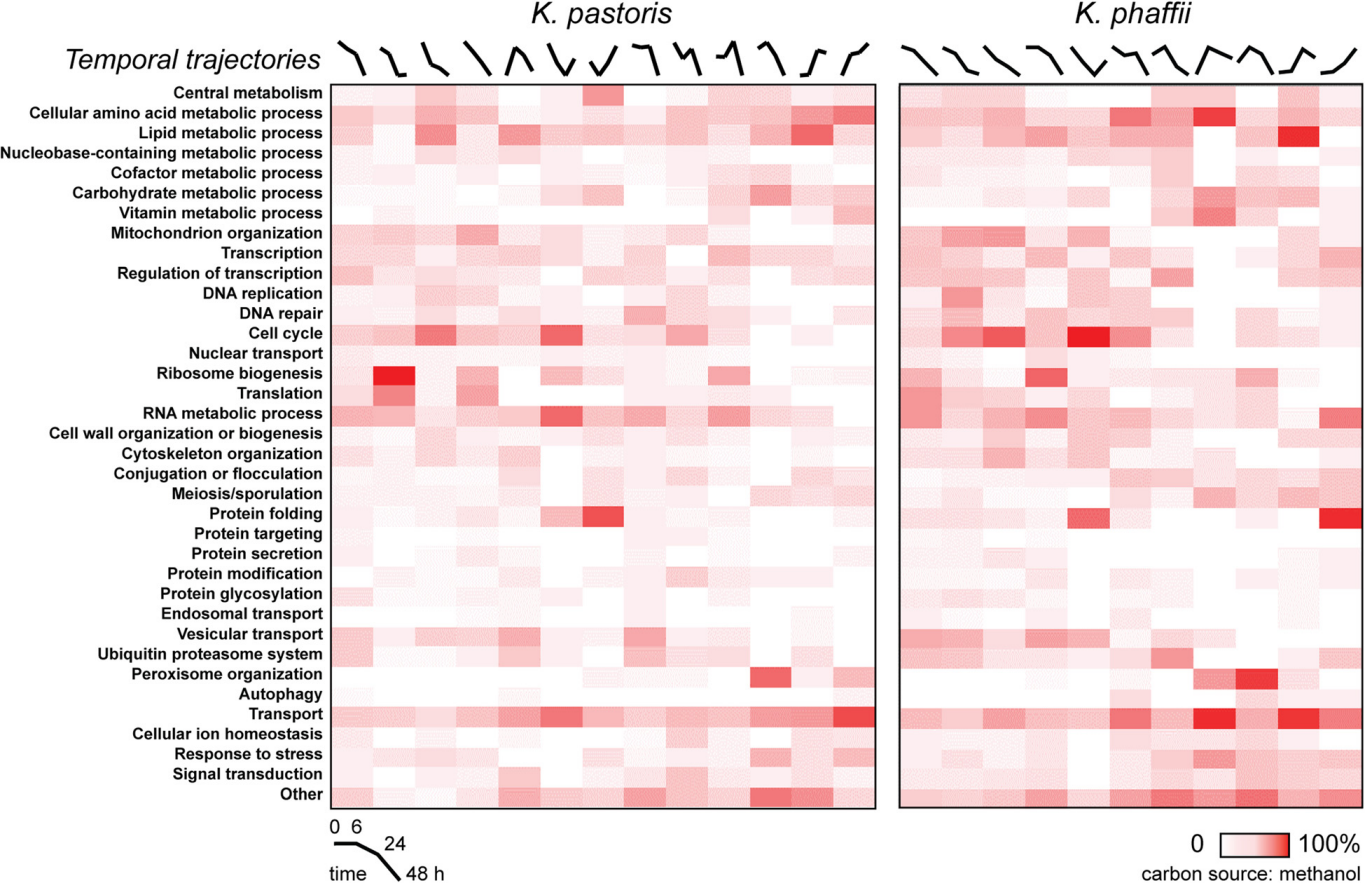
Biological process enrichment as a function of cultivation in methanol. Heat map representation of the enrichment of GO biological process terms for expression phenotypes observed in *K. pastoris* and *K. phaffii* during a 48 h batch cultivation in methanol as characterized by self-organizing maps (SOMs). Representative temporal trajectories of gene expression were generated for each SOM by averaging expression data at each time point for genes present within a given map. Color density relates to the number of genes assigned to a particular process as a percentage of the total number genes present in a particular expression phenotype or map

To better understand how particular cellular processes may associate with the expression phenotypes, we used simplified GO biological process terms [39] to classify all genes of known function present in the analysis into 36 distinct groups (Additional file 10: Table S9). Pathway associations for expression phenotypes were largely similar between the two *Komagatella* species (Fig. 5, Additional file 2: Figures S11 and S12). Transport-related genes were associated with phenotypes wherein gene expression increased during the cultivation period; these phenotypes were also dominated by metabolic processes, which could imply coordinated transcription. Genes related to translation and protein expression were strongly correlated with decreasing expression over time and are inversely correlated with phenotypes indicating increased gene expression over time, particularly for glycerol and methanol growth conditions. These results likely indicate that the cultures are approaching stationary phase at the end of cultivation sampling, as similar results of decreased gene expression have been reported as cellular growth rates decline [40]. Protein folding machinery also generally increased in expression over time, (with the notable exception in the glycerol cultivation of *K. phaffii*), likely due to the triggering of stress responses as cultivation progresses. Indeed, we observed that HAC1, a master stress response regulator [41], is a highly differentially expressed gene during cultivation in methanol. Secretory pathway-resident genes are inversely correlated with increasing expression phenotypes, particularly in glycerol and methanol. These results together suggest that optimizing *Komagatella* strains as expression hosts for sustained protein expression and secretory function during cultivation will require genome engineering and concomitant optimization of fermentation.

### Characterization of *Komagatella* secretome

We were intrigued by the decline in secretory function over time observed in our expression analysis. We postulated that this result could stem from a decline in the expression of proteins entering the secretory pathway, which could have beneficial implications for simple downstream purification of material produced in *Komagatella*. Previous characterization of the secretome expressed in *K. pastoris* highlighted its utility as a host organism in producing minimally contaminated heterologous proteins when cultivated in glucose [13]. We identified 170 1:1 orthologs predicted to have a signal peptide present in both strains. Single sample Gene Set Enrichment Analysis (ssGSEA) [42] was used to compare the expression of these genes between the two strains in each carbon source cultivation condition (Additional file 2: Figure S13A). While it appears that *K. pastoris* had elevated secretory protein expression compared to *K. phaffii*, these proteins trended downward in expression in both strains over time, which we verified by examination of cultivation supernatants using SDS-PAGE (Additional file 2: Figure S13B). Our data suggests that cultivation of *K. phaffii* in methanol has the greatest potential to yield a cultivation supernatant with few contaminating host cell proteins, as the overall expression of the secretome is lowest for this condition.

### Characterization of mutational variation in *K. phaffii* GS115

**Fig. 6:**
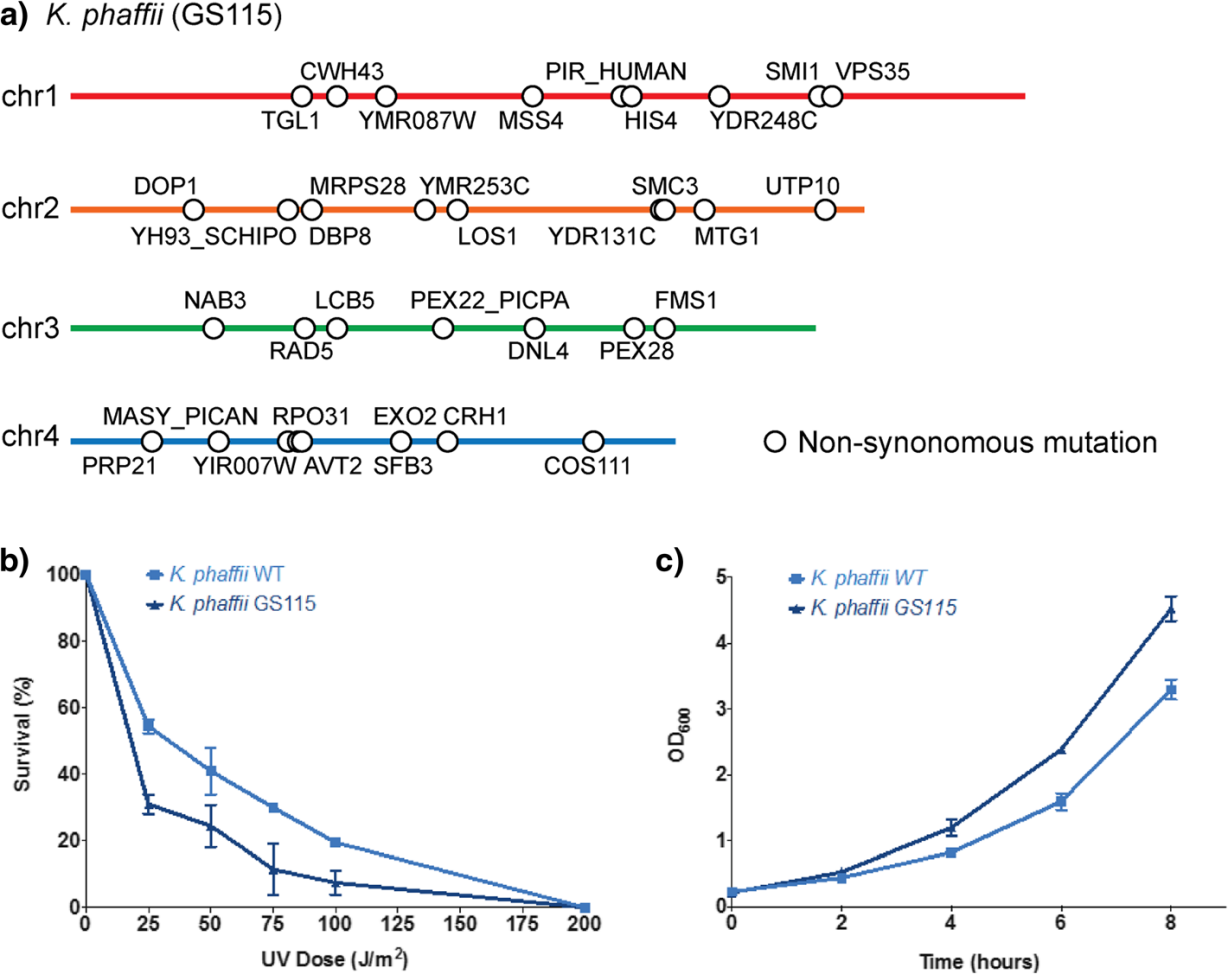
Locations of mutations found in GS115 and phenotypic differences observed between GS115 and wildtype *K. phaffii*. **a) ** The chromosomal locations of the 35 single nucleotide polymorphisms (SNPs) found in GS115 relative to wildtype *K. phaffii*. **b) ** Growth curve of *Komagatella* strains on glucose media. Wildtype *K. phaffii* growth data is indicated with squares and GS115 growth data is indicated with triangles. Data shown for each strain is the mean from triplicate measurements. Error bars indicate 95 % confidence intervals. **c** Kill curve of *Komagatella* strains following exposure to UV light. Data shown for each strain is the mean from two experiments, each run in triplicate. Error bars are the standard deviation across all data collected for both experiments

Given the exceptional coverage of the *K. phaffii* genomes, we also characterized the point mutations present in the GS115 strain. This derivative strain was selected for histidine auxotrophy following random mutagenesis with nitrosoguanidine [11]. Beyond the known mutation in the HIS4 gene, no description of the mutations present in this organism has been reported. We found 35 single nucleotide polymorphisms (SNPs) with potential influence to protein function randomly distributed across the four major chromosomes in the GS115 strain (Fig. 6a and Additional file 11: Table S10), out of which 32 non-synonymous mutations were in coding regions, one was in a 3′ untranslated region (UTR) and the remaining two were the gain and loss of a stop codon respectively (Additional file 12: Table S11). No other types of mutations, including indels or GCRs, were detected.

We examined the potential of detected mutations to impact the organism’s phenotype *in silico*. First, the genes containing mutations were annotated for existence of known functional domains. Then, a conservation-based evaluation of the impact of each mutation on protein function was performed. Several mutations were predicted to strongly impact protein function due to mutation of highly conserved amino acid residues within essential domains. One notable example in the HIS4 gene was the C557R mutation that occurs in the histidinol_dh domain and is responsible for gene inactivation and strain auxotrophy. Several mutations lie outside protein domains, but are still predicted to impact protein function, including the S752F mutation in the DNA repair protein RAD5 (radiation sensitivity protein 5). We compared the survival rates of WT phaffii and GS115 to increasing levels of UV radiation and noted that GS115 displayed lower survival rates overall (Fig. 6b), indicating a phenotypic difference that could be related to the point mutation in RAD5 [43].

Finally, the expression of each of the 35 mutated genes was also directly examined by comparing gene expression between WT and GS115 for each time point sampled during a cultivation (10 possible comparisons). Gene YDR248C—a probable gluconokinase (http://www.uniprot.org/uniprot/Q03786) that is connected to the pentose phosphate pathway (PPP) [44]—consistently displayed lower expression in GS115 relative to wild type in six out of the ten conditional expression comparisons. Given the role of the PPP in amino acid precursor formation and its complex relationship with glycolysis [45], we postulated that there could be a difference in doubling time between the two strains. Indeed, the GS115 consistently outgrew the WT strain (Fig. 6c). These comparisons between wildtype *K. phaffii* and GS115 suggest that many of these discovered mutations may have direct phenotypic effects; based on our conservation and expression analyses as many as half of the reported mutations could be consequential to phenotype and each one should be studied independently.

## Conclusions

Here, we have established a comprehensive foundation for both the genomes and transcriptomes of the two organisms that comprise *Pichia pastoris*. The refined genomic sequences and assemblies now enable direct comparisons of both organisms and establish a base for specific engineering of each one. The transcriptomic analyses from RNA-sequencing of batch cultivations for each strain in three common carbon sources provide a well-defined reference from which further understanding of metabolism and heterologous gene expression can be derived. These data reveal interesting opportunities for improved selectivity of expression, novel sites for integration, and a framework for metabolic modeling and engineering. There remain many interesting elements to explore, including the inter-relationships between locus accessibility and promoter activity on gene expression under different carbon sources. The discovery of telomeric and linear plasmid sequences should facilitate the engineering of new vectors or artificial chromosomes. The insights to the organism’s transcriptional activity should inform both host engineering and process engineering for biologic production. For example, the reduced burden of host cell proteins with time highlights an attractive feature of this host for subsequent purification of products. The detailed mapping of mutations in the GS115 strain will help guide the intentional engineering of enhanced hosts with specific phenotypic benefits, such as enhanced growth. The common ground provided here can now enable systematic efforts to understand the genetic basis of enhanced protein expression in optimized strains and generate mechanistic insight into the cell biology of *P. pastoris*. In turn, these advances ultimately will improve the productivity and robustness of an increasingly important host for the global manufacturing of protein biologic drugs.

## Methods

### Genome sequencing

The three *Komagataella* strains - (1) *K. pastoris* (NRRL Y-1603 or ATCC 28485), (2) *K. phaffii* ‘WT’ (NRRL Y-11430 or ATCC 76273) and (3) *K. phaffii* GS115 (ATCC 20864) were grown overnight in YPD (BD Difco, Cat. # 242820). DNA was extracted using the YeaStar Genomic DNA Kit (Zymo Research, Cat. # D2002) and RNA using the YeaStar™ RNA Kit (Zymo Research, Cat. # R1002). The extracted DNA was sequenced on the Pacific Biosciences (PacBio) single molecule real-time (SMRT) platform. Genomic DNA was also sequenced on Illumina HiSeq2000 from both fragment and jumping libraries. Illumina fragment libraries were generated as previously described [46] with the following modifications. For each sample, 100 ng of genomic DNA was sheared to 200 bp in size using a Covaris LE220 instrument (Covaris, MA) with the following parameters: temperature: 7–9 °C; duty cycle: 20 %; intensity: 5; cycles per burst: 200; time: 90 s; shearing tubes: Crimp Cap microTUBES with AFA fibers Covaris, MA). DNA fragments were end repaired, 3′ adenylated, ligated with indexed Illumina sequencing adapter, and PCR enriched, as previously described [47]. The resulting Illumina fragment sequencing libraries were normalized and were size selected to contain inserts of 180 bp ±3 % in length using a Pippen Prep system (Sage Science, MA) following the manufacturer’s recommendations. In jumping libraries, JUMP processing deletes the DNA in between the sections of interest that are far apart and combines them in order to be sequenced. Initial genomic DNA was sheared to get the sample to 5 kb in 150 ul. A caliper quality check was performed after end repair to insure proper shearing, and a critical circularization step was performed for 16 h. A second shear was performed to lower the size of the DNA to 500 bp after an exonuclease cleanup. Immobilization, a second end repair, an A-base addition, and PCR was performed for 18 cycles, which were all followed by washes. Adaptor ligation with Illumina paired end adapters also was performed before PCR to ensure the samples can be pooled before being sequenced. The multiple wash steps ensures clean PCR product is being loaded on sequencers.

Illumina sequencing libraries were quantified using quantitative PCR (KAPA Biosystems, MA) following the manufacturer’s recommendations. Libraries were normalized to 2 nM and denatured using 0.1 N NaOH.

Sequencing Flowcell cluster amplification was performed according to the manufacturer’s recommendations using the V3 TruSeq PE Cluster Kit and V3 TruSeq Flowcells (Illumina, CA). Flowcells were sequenced with 101 base paired end reads on an Illumina HiSeq2000 instrument, using V3 TruSeq Sequencing by synthesis kits and analyzed with the Illumina RTA v1.12 pipeline (Illumina, CA).

### Genome assembly

Pacbio reads were assembled using a hierarchical genome-assembly process (HGAP) [48] and the Illumina paired-end reads were aligned [49] to the assemblies for error correction and assembly improvement using Pilon [50]. Assemblies were refined by manual curation. Pairwise alignments were carried out using BLAST (version 2.2.27). For each genome assembly, contigs were examined and removed if redundant (i.e. aligning to any other contig in the same assembly with >90 % identity). All contigs containing rDNA repeats were excluded from the above step. Large contigs were manually connected to construct telomere-to-telomere sequences and checked for consistency with the previously reported genome [12]. One gap in the wildtype *K. phaffii* assembly was closed by using corresponding and overlapping sequence from *K. phaffii* GS115 to bridge the missing segment. The validity of this bridging process was supported by manual examination of PacBio and Illumina sequences and raw sequencing reads that documented the manual junctions. The genomic sequencing data and assembled and annotated genomes are deposited at NCBI under bioproject accession numbers PRJNA304627 (*K. pastoris*), PRJNA304977 (*K. phaffii* wildtype), and PRJNA304986 (*K. phaffii* GS115).

### Transcriptome analysis

The three Komagatella strains were grown in shake flask (30 °C, 250 rpm, μ_avg_ = 0.26) using complex glycerol-containing media (BMGY, Teknova, Cat. # B8000) to an OD_600_ of 2.0 (low density) or to an OD_600_ of 20 (high density). Following initial biomass accumulation, the cells were harvested by centrifugation and re-suspended in either glycerol- (BMGY), glucose- (YPD, BD Difco, Cat. # 242820) or methanol-containing media (BMMY, Teknova, Cat. # B8100). Samples were collected before changing media (0 h) and after resuspension into fresh media (6, 24 and 48 h). RNA was extracted from three independent cultivations for each time point sampled using the RNAeasy Kit (Qiagen, Cat. # 74104) and analyzed to ensure that RNA Integrity Number (RIN) score was >7. RNA sequencing libraries were constructed using the Truseq mRNA stranded HT kit (Illumina, Cat. # RS-122-2103) and sequenced on the Illumina NextSeq platform to generate 75-nucleotide paired-end reads at a read depth of at least 3 million reads per sample.

To assess the technical quality of RNA-seq reads for each condition sampled, each raw data set was down-sampled to 1 M paired-end reads and aligned to the assembly using BWA 0.7.5a. Then, Bedtools (version 2.17.0) was used to overlap the resulting alignments to the annotations to count the reads falling into genes, coding regions, intronic regions, 5′ or 3′ UTRs, flanking 3 kb genic regions and intergenic regions. Other basic statistics, including mapping rate, unique mapping rate, multiple mapping rate, number of perfect match reads, number of alignments with 1 or 2 mismatches and ratio of sense vs. anti-sense reads were also collected for each sample (see Additional file 3, Table S2 with quality control data for each RNA-seq sample). The complete data set set for each condition and time point was used for all analyses reported. RNA-seq data are deposited at NCBI under the bioproject accession number PRJNA304627.

### Copy number analysis

DNA sequencing reads were down-sampled to ~2 M reads for both *K. pastoris* and wildtype *K. phaffii* WT samples; HMMcopy (version 0.1.1) was used to evaluate the copy number in 1000 bp windows. Mappability and GC content tracks were generated as control following the HMMcopy documentation. BWA (version 0.7.10) was used to map the reads to the reference (default options were used).

### Genome annotation

For initial annotation purposes, the standard fungal annotation pipeline used by the Broad Institute Genome Sequencing Platform [22] was deployed on these genomes. Given the low proportion of spliced genes (<5 %), protein-coding gene predictions were more accurately made using a prokaryotic *ab initio* tool, thus Prodigal [51] was employed. The rRNA loci were predicted by RNAmmer (version 1.2) [52] and tRNA by tRNAscan-SE (version 1.12) [53]. RNA was also sequenced on the Illumina platform (see above) and a subset of the resulting RNA-seq reads were assembled using Trinity (version r20140717) [54] and aligned to the genomes. PASA [22] was then run using the Trinity assembly alignments to update the prodigal genesets with splicing and UTR information. The genes were then filtered to remove all genes smaller than 300 nucleotides without evidence of overlap to either Hmmer, GeneWise (version 2.2.0) or RNA-seq data. Genes were then repeat-filtered using an in-house repeat-filtering pipeline (TPSI: e-value of 1e-10 and a minimum of 30 % Query Coverage; RepBase; repeat Hmmer domains; and Multihits: > = 8 times without non-repeat domains, minLength = 100, and percentId > = 90 %). Quality control was performed and genes were repaired so that none contained partial codons, in-frame stops, or unknown bases. Finally, protein-coding genes demonstrating 70 % overlap with non-coding genes (rRNA or tRNA annotations) were filtered out. Gene product was assigned based on BLAST best hit (E-value < 1e-10, version 2.2.25) against three databases in the following order of precedence: i) Swissprot (release 2011_03; at > =60 % identity and > =60 % query coverage, <30 % length difference), ii) TIGRfam (TIGRfam13), iii) KEGG (version 65; at > =60 % identity and > =60 % query coverage, and must have KO#). Genes without a match had their product defined as “hypothetical proteins”. Following initial gene identification, unmapped and intergenic RNA-seq reads from all culture conditions were subjected to a second Trinity assembly in an effort to identify conditionally dependent novel transcripts. The resulting transcripts from this second trinity run were included in subsequent annotation and gene expression analyses.

The genome annotations for each of the three strains were linked to the two other publicly-banked complete *K. phaffii* genomes with annotations [12, 14]. The refseq proteins from GS115_644223 and all proteins from CBS7435_981350 (both banked with NCBI) were used as separate BLAST (version 2.2.27) targets for all proteins from our three genomes. The best BLAST hit, as defined by highest bit score, per protein are reported for each genome (Additional file 4, Table S3).

### Orthology assignment and gene naming

Sequence clustering at the mRNA level was used to identify orthologs between strains. For each pairwise strain comparison, mRNA sequences were pooled into a FASTA file and used as input for the application CD-HIT-EST (version 4.6.1) [55]. For comparison of orthology in wildtype *K. phaffii* versus *K. phaffii* GS115, a clustering threshold of 95 % identity was used. For comparison of orthology in *K. pastoris* versus *K. phaffii*, a lower clustering threshold of 80 % identity was used due to divergence between strains. Orthology percent identity analysis was derived from CD-HIT clustering results using custom scripts.

This automated analysis resulted in identification of 4,601 orthologous pairs between *K. pastoris* and *K. phaffii*. Manual analysis of the clustering using a custom script, TIBCO Spotfire and Integrated Genomics Viewer (version 2.3.72) resulted in annotation of an additional 48 ortholog pairs. Of the 595 remaining sequence clusters with unclear orthology, 33 contain only Trinity transcript groups of varying complexity. 129 have complex relationships, such as 2:1 (91 examples) or 2:2 (38 examples), typically caused by gene prediction artifacts during annotation of adjacent genes, including fragmented gene prediction or incomplete UTR annotation. 7 genes found in *K. phaffii* are not found in *K. pastoris*, those located on the linear plasmid. 398 clusters contain a single sequence that cannot be further associated by clustering at lower threshold and may represent species-specific genes. To address the possibility that these genes result from data contamination from other organisms, these 398 proteins were used to query Swissprot with BLASTP (2.2.27), but we observe no high-identity matches to any known proteins. The resulting alignments are consistent with species-specific genes, usually of fungal origin, rather than contamination with genetic material from other species.

For wildtype *K. phaffii* and *K. phaffii* GS115, 4,996 ortholog pairs were identified from the 5,169 sequence clusters by the automated analysis described above. (Note: the 5,169 sequence clusters do not correspond to genes, but are collections of related sequences.) Of the remaining 163 sequence clusters with unclear orthology, 30 contain only Trinity transcript groups of varying complexity and 46 clusters have complex relationships, such as 2:1 (25 examples), 2:2 (20 examples), or 3:2 (1 example). Forty-seven sequence clusters are found in wildtype, but not GS115, including the seven genes encoded on the linear plasmid; 40 clusters are found in GS115 but not wildtype. 50 of these 80 sequence clusters form 25 pairs of orthologs when a lower identity threshold is used during clustering with CD-HIT (85 % instead of 95 %). To address the remaining 30 sequences, TBLASTN (version 2.2.27) analysis was performed using proteins found in one strain to query the genome of the other strain. These analyses suggest that 1 wildtype genes and 13 GS115 genes are present in the other strain despite the lack of gene prediction during annotation. 1 additional wildtype gene (GQ67_00697) that is situated in a complex locus was unannotated in GS115, though underlying nucleotide sequences in the strains align with high identity. Alignment between the predicted protein from wildtype gene GQ67_04936 and the DNA sequence from GS115 indicates a frame shift; this gene may by inactivated in GS115. GS115 genes GQ68_05325 and GQ68_05326 are located near the terminus of Chr 4 in that strain, but a neighboring gene (GQ68_05329) has an ortholog ~200 Kb from the end of Chr 4 in wildtype indicating a small gene rearrangement between the strains.

Additional analysis with Kraken [56] (version 0.10.6) and the MiniKraken database, consisting of bacterial, archaeal and viral genomes, demonstrated a low degree of sequence contamination in our genomic reads at 0.19 % in *K. phaffi* WT, 0.37 % in *K. phaffi* GS115 and 3.23 % in *K. pastoris,* with the majority of contaminating sequences matching bacterial taxonomies. BLASTX (2.2.27) alignment of potentially contaminating reads to the predicted proteins from all 3 of our genomes yielded shortened or low identity results, indicating that these reads are non-identical to any predicted proteins in the genome annotations. These results indicate that sequence contamination does not contribute to gene prediction and annotation, nor to the sequence sets that lack clear orthology between strains.

*Komagataella* genes were associated with *S. cerevisiae* genes using BLAST-based approaches (version 2.2.27). The results were parsed and best hits were compared using a combination of custom scripts, Tibco Spotfire (version 6.5.3.12) and MySQL. *Komagataella* orthologs with consistent reciprocal best hits to *S. cerevisiae* genes were named according to the *S. cerevisiae* convention. Three thousand five hundred sixty-six *Komagataella* ortholog groups were thus named with *S. cerevisiae* gene names. An additional 30 genes associated either with flocculation [25], or central carbon metabolism [14, 26] (including the methanol utilization (MUT) pathway) were manually assigned, though these genes may not correspond to *S. cerevisiae* (see Additional file 5: Table S4 for a complete list of named orthologs).

### Phylogenetic analysis

Multiple sequence alignment was carried out using alignments derived from ten randomly selected proteins found in all three *Komagataella strains* and with apparent 1:1 orthology relationships in any pairwise comparison between species. After gaps were removed, the phylogeny was constructed using a concatenated alignment and reliability was assessed using bootstrapping. Phylogenetic trees were calculated with neighbor-joining, distance-based, maximum likelihood and maximum parsimony methods using the Phylip (version 3.6.96) package of programs. The highest confidence clades having 100 % bootstrap support in all methods were highlighted.

### Codon usage

Codon usage was determined using ANACONDA (version 2.0.1.15). The coding sequences were extracted from the genome annotations and used as input for ANACONDA. ANACONDA determines codon usage as Relative Synonymous Codon Usage (RSCU).

### Gene expression analysis

RNA-seq analysis was performed using RSEM (version 1.2.15) with bowtie2 (version 2.2.3) and a transcriptome alignment target containing a combination of original annotated transcripts plus transcripts derived from *de novo* Trinity assembly. Gene and isoform FPKM and count data were processed for analysis with custom scripts and Tibco Spotfire (version 6.5.3.12). Count data for differential expression testing was done using DESeq (version 1.10.1) and R (version 2.15.3). Unsupervised hierarchical clustering (Ward’s Method) was used to examine data relationships for the three biological replicates performed for each cultivation condition. In all but 3 cases, consistent clustering of biological replicates were observed. For those 3 cases, (wildtype *K. phaffii*, 48 h Glucose Replicate 1; wildtype *K. phaffii*, 24 h Glycerol Replicate 1; and *K. phaffii* GS115, 48 h Glucose Replicate 3) the inconsistent replicate was excluded from downstream analyses. A table of raw expression data (log2 FPKM and integer count) for all genes and replicates included in the analyses of each of the three genomes is provided (Additional file 13: Table S12).

For a subset of cultivation conditions, RNA-seq was performed using RNA collected from a higher density cultivation (see above). Unsupervised hierarchical clustering suggested expression data were highly correlated between similar cultivation conditions sampled at two different densities. To confirm this correlation, a custom R script called bivariatetrelliscompact. R was prepared to calculate Pearson correlation coefficients between vectors of gene expression data for each strain, culture, and density condition. The input vectors were averages of 3 biological replicates from the initial Tophat-based processing of the data. The correlation coefficients obtained range between 0.969 and 0.995 (Additional file 2: Figure S14).

### Alternative splicing analysis

Alternatively spliced isoforms that result in alterations to protein coding sequences were identified in the initial gene annotation using Bedtools (version 2.20.1); the expression values associated with these isoforms was extracted from the RSEM output (see above). Results were used for downstream expression analysis of alternatively spliced genes in various cultivation conditions. In order to address the scale of uncaptured alternative spliced isoforms, reads from two diverse low-density cultivations (Glycerol, 0 h and Methanol, 48 h) were aligned to the corresponded genomes using Tophat (version 2.0.12), allowing for novel exon junction discovery. Novel junctions that overlapped coding sequences and had >5 distinct supporting reads were identified using custom scripts and Bedtools (version 2.20.1). We defined this additional resulting exon-junction set as containing “potential CDS changing junctions” (Additional file 7: Table S6).

### Linear plasmid mismatch rate analysis

PacBio reads from each strain were aligned to the linear plasmid found in wildtype *K. phaffii* with bwa-sw (version 0.7.10). Illumina reads were aligned with bwa-mem (version 0.7.10) and mismatch rates per 1000 bases per 1000 reads were calculated with custom scripts.

### Analysis of expression data sampled from batch cultivation using Self Organizing Maps (SOMs)

Gene expression data processing was guided by previous methods [38]. A low-expression, low-variance filter was implemented to exclude genes that may not be expressed. Genes with average log2FPKM <1 and variance <0.5 across the 10 cultivation condition averages were excluded. No condition-specific fold change filter was implemented. The GenePattern module PreprocessDataset was used to row normalize the data by setting averages to 0 and variances to 1. The preprocessed data were then used as input to the GenePattern module SOMClustering with a cluster range of 2–50. Elbow analysis was performed to identify the optimal number of clusters that minimizes degeneracy, where the variance captured by additional clusters was less than 0.01. The corresponding odf file was selected for additional analysis. Representative profiles for each map were generated by averaging expression data at each time point within a given map.

### Secretome identification and analysis

Potential secreted proteins were identified using Signalp (version 4.1) and custom processing scripts. Genes with a predicted signal peptide and having clear orthologs in all strains were used to create a gene set for single sample Gene Set Enrichment Analysis (ssGSEA) [42]. ssGSEA was performed using R (version 3.2.2) and scripts freely downloaded from the Broad GenePattern server (http://genepattern.broadinstitute.org/). The resulting ssGSEA projections were normalized using PreprocessDataset.

### Mutational variant calling

Variant calling was carried out using the GATK Best Practices workflow [57]. Alignments were performed using Burrows-Wheeler Aligner (BWA-MEM, version 0.7.5a) [49]. *K. phaffii* GS115 Illumina sequencing reads were mapped to the wildtype *K. phaffii* PacBio genome assembly and wildtype *K. phaffii* Illumina sequencing reads were mapped to the *K. phaffii* GS115 PacBio genome assembly. PCR duplicates were removed using Picard (version 1.94) MarkDuplicates after sorting the sequences using SortSam. Samtools (version 0.1.19) was used for the first round of SNP and Indel calling. These high quality Indels and SNPs were then selected as the input for GATK Best Practice Indel local realignment and base quality recalibration steps (version 3.1.1). Variation calling output by GATK was annotated to identify non-synonymous SNPs using Snpeff (version 2.0.5d, http://snpeff.sourceforge.net) (Additional file 12: Table S11). This larger set of variations was further refined to a list of high confidence variations by identifying reciprocal genotype calls in both strains.

The potential function of these mutations was characterized in more detail using a two-fold approach. First, the variants were annotated with respect to protein domain using SMART (http://smart.embl-heidelberg.de/) and Pfam (http://pfam.xfam.org/). Second, conservation-based evaluation of the impact of the observed amino acid substitutions were scored using SIFT (http://sift.jcvi.org/). The reference orthologous protein sequences for use in SIFT analysis were obtained from the Fungal Orthogroups Repository (http://www.broadinstitute.org/regev/orthogroups/).

## Additional files

Additional file 1: Table S1. Genome sequencing, assembly and annotation statistics. (XLSX 13 kb) Additional file 2: Supplementary figures. **Figure S1.** Experimental timeline for RNA sequencing of *Komagatella* strains. Schematic timeline for collection of RNA samples during batch cultivation of strains in shake flasks on three different carbon sources. Three independent cultivations were sampled for each time point. **Figure S2.** Phylogenetic comparison of *K. pastoris* and *K. phaffii* to other related yeasts. Phylogeny was generated using a concatenated, gap-free alignment of ten orthologous proteins. Phylogenetic tree was calculated using neighbor-joining, distance-based, maximum likelihood and maximum parsimony methods; reliability was assessed using bootstrapping. Clades marked with an asterisk are supported by 100 % of bootstrap replicates in all four methods. **Figure S3.** Gene conservation between *K. pastoris* and *K. phaffii*. a) Histogram denoting homology at the base pair level for all 1:1 orthologous genes. b) Alignment of the P_GAPDH_ promoter element between *K. pastoris* and *K. phaffii*. c) Alignment of the P_AOX1_ promoter element between *K. pastoris* and *K. phaffii*. **Figure S4.** Copy number determination for major chromosomes in a) *K. pastoris*, b) *K. phaffii* wild-type and c) *K. phaffii* GS115 strains. **Figure S5.** Codon usage for a) *K. pastoris* and b) *K. phaffii* as determined from all coding sequences identified in genome annotation. The relative abundance observed for each codon is represented as a percentage of total codon usage for the corresponding amino acid. **Figure S6.** Isoform expression in *K. pastoris* and *K. phaffii* as a function of cultivation conditions. Heat maps of gene expression (log2 fpkm) for isoforms of alternatively spliced genes that alter coding sequences in a) *K. pastoris* and b) *K. phaffii* detected in initial genome annotation. Alternatively spliced genes with sufficient homology to *S. cerevisiae* are named, otherwise gene identifiers from genome annotation are used. Isoform expression is shown as a function of batch growth in glycerol, glucose or methanol during a 48 h cultivation period. **Figure S7.** Chromosomal locations of highly expressed genes. Map of chromosomal location (base pair identity) for the most highly expressed genes (top 10 % expression) in a) *K. pastoris* and b) *K. phaffii*. Black lines indicate gene expression level at 24 h time points during batch cultivation in either glycerol, glucose or methanol. Red lines indicate locations of GC-rich autonomously replicating sequence (GC-ARS) motifs identified by BLAST. **Figure S8.** Variance in gene expression during batch cultivation of *K. pastoris* and *K. phaffii*. Scatter plots of the average variance versus expression observed for all annotated genes across the 10 conditional averages generated from either a) *K. pastoris* or b) *K. phaffii* expression data. Genes with average log2 fpkm <1 and variance <0.05 were excluded from further analyses. c) Elbow analysis of input cluster number to identify optimal expression data clustering by self- organizing maps (SOMs). Large circles denote the number of clusters for each expression data set where the additional variance captured by further clustering was < 1 %. **Figure S9.** Gene expression phenotypes in *K. pastoris* as a function of cultivation conditions. Self-organizing maps (SOMs) of genes changing expression similarly in *K. pastoris* during a 48 h batch cultivation in a) glycerol, b) glucose or c) methanol. **Figure S10.** Gene expression phenotypes in *K. phaffii* as a function of cultivation conditions. Self-organizing maps (SOMs) of genes changing expression similarly in *K. phaffii* during a 48 h batch cultivation in a) glycerol, b) glucose or c) methanol. **Figure S11.** Biological process enrichment as a function of cultivation in glucose. Heat map representation of the enrichment of GO biological process terms for expression phenotypes observed in *K. pastoris* and *K. phaffii* during a 48 h batch cultivation in glucose as characterized by self-organizing maps (SOMs). Representative temporal trajectories of gene expression were generated for each SOM by averaging expression data at each time point for genes present within a given map. Color density relates to the number of genes assigned to a particular process as a percentage of the total number genes present in a particular expression phenotype or map. **Figure S12.** Biological process enrichment as a function of cultivation in glycerol. Heat map representation of the enrichment of GO biological process terms for expression phenotypes observed in *K. pastoris* and *K. phaffii* during a 48 h batch cultivation in glycerol as characterized by self-organizing maps (SOMs). Representative temporal trajectories of gene expression were generated for each SOM by averaging expression data at each time point for genes present within a given map. Color density relates to the number of genes assigned to a particular process as a percentage of the total number genes present in a particular expression phenotype or map. **Figure S13.** Secretory pathway protein expression in *K. pastoris* and *K. phaffii*. a) Row normalized single set Gene Set Enrichment Analysis (ssGSEA) projections for 170 proteins bearing a signal peptide as identified by Signalp. b) SDS-PAGE analysis of host-cell protein expression in supernantants during batch cultivation of *K. pastoris* and *K. phaffii* for 48 h in glucose or glycerol-containing media. **Figure S14.** Correlation of gene expression at two different cultivation densities. Scatter plots of gene expression between similar cultivation conditions for a) *K. pastoris*, b) wildtype *K. phaffii*, and c) *K. phaffii* GS115 grown at two different cell densities. Density A corresponds to cultures outgrown to OD_600_ = 2.0 prior to sampling and Density B corresponds to cultures outgrown to OD_600_ = 20 prior to sampling. Pearson correlation coefficients were calculated from expression vectors that were averages of three biological replicates for each cultivation condition and density. (PDF 1991 kb) Additional file 3: Table S2. RNA sequencing statistics. (XLSX 31 kb) Additional file 4: Table S3. BLAST-based linkage of reported genome annotations to existing annotations available for *K. phaffii* strains. (XLSX 3291 kb) Additional file 5: Table S4. Gene names for simple orthologs (1:1:1 association between *Komagataella* species) given by association with *S. cerevisiae* or manually assigned. (XLSX 148 kb) Additional file 6: Table S5. Linear plasmid sequencing statistics. (XLSX 9 kb) Additional file 7: Table S6. Alternatively spliced genes in *K. pastoris* and *K. phaffii*. (XLSX 25 kb) Additional file 8: Table S7. GC-ARS motif locations in *K. pastoris* and *K. phaffii*. (XLSX 30 kb) Additional file 9: Table S8. GO annotation of genes that are highly and differentially expressed in a particular carbon source during fermentation. Genes classified here were highly (top 10 % expression) and differentially expressed (log2-fold change >2, *p* < 0.05) during fermentation on the carbon source listed. (PDF 393 kb) Additional file 10: Table S9. Self-organizing maps (SOMs) of expression phenotypes observed in *K. pastoris* or *K. phaffii* and annotated with simplified GO biological process terms. (XLSX 5464 kb) Additional file 11: Table S10. List of mutational variants in GS115 and their potential to affect protein function. Lower SIFT scores indicate higher degrees of conservation for the native amino acid affected by the nucleotide change. Scores below the default threshold of 0.05 are highly likely to affect protein function. The differential expression summary column consists of three numbers in x/y/z format, where x corresponds to the number of culture conditions (out of 10 total) where the gene is found to be significantly differentially expressed between GS115 and wildtype *K. phaffii* (log fold change >2, *p* <0.05), y is the number of times the expression comparison tests are significantly higher in wildtype *K. phaffii*, and z is the number of times the expression comparison test are significantly higher in GS115. (PDF 339 kb) Additional file 12: Table S11. Summary of mutational variants found in GS115. (PDF 176 kb) Additional file 13: Table S12. Raw gene expression data for all replicates, all genes (log2 FPKM and integer counts). (XLSX 8921 kb)

## References

[1] Walsh G (2010). Biopharmaceutical benchmarks 2010. Nat Biotechnol.

[2] Aggarwal RS (2014). What’s fueling the biotech engine-2012 to 2013. Nat Biotechnol.

[3] Corchero JL, Gasser B, Resina D, Smith W, Parrilli E, Vazquez F, Abasolo I, Giuliani M, Jantti J, Ferrer P (2013). Unconventional microbial systems for the cost-efficient production of high-quality protein therapeutics. Biotechnol Adv.

[4] Love JC, Love KR, Barone PW (2013). Enabling global access to high-quality biopharmaceuticals. Curr Opin Chem Eng.

[5] Ahmad M, Hirz M, Pichler H, Schwab H (2014). Protein expression in Pichia pastoris: recent achievements and perspectives for heterologous protein production. Appl Microbiol Biotechnol.

[6] Shekhar C (2008). Pichia power: India’s biotech industry puts unconventional yeast to work. Chem Biol.

[7] Meehl MA, Stadheim TA (2014). Biopharmaceutical discovery and production in yeast. Curr Opin Biotechnol.

[8] Maccani A, Landes N, Stadlmayr G, Maresch D, Leitner C, Maurer M, Gasser B, Ernst W, Kunert R, Mattanovich D (2014). Pichia pastoris secretes recombinant proteins less efficiently than Chinese hamster ovary cells but allows higher space-time yields for less complex proteins. Biotechnol J.

[9] Kurtzman CP (2005). Description of Komagataella phaffii sp. nov. and the transfer of Pichia pseudopastoris to the methylotrophic yeast genus Komagataella. Int J Syst Evol Microbiol.

[10] Kurtzman CP (2009). Biotechnological strains of Komagataella (Pichia) pastoris are Komagataella phaffii as determined from multigene sequence analysis. J Ind Microbiol Biotechnol.

[11] Cregg JM, Barringer KJ, Hessler AY, Madden KR (1985). Pichia pastoris as a host system for transformations. Mol Cell Biol.

[12] De Schutter K, Lin YC, Tiels P, Van Hecke A, Glinka S, Weber-Lehmann J, Rouze P, Van de Peer Y, Callewaert N (2009). Genome sequence of the recombinant protein production host Pichia pastoris. Nat Biotechnol.

[13] Mattanovich D, Graf A, Stadlmann J, Dragosits M, Redl A, Maurer M, Kleinheinz M, Sauer M, Altmann F, Gasser B (2009). Genome, secretome and glucose transport highlight unique features of the protein production host Pichia pastoris. Microb Cell Fact.

[14] Kuberl A, Schneider J, Thallinger GG, Anderl I, Wibberg D, Hajek T, Jaenicke S, Brinkrolf K, Goesmann A, Szczepanowski R (2011). High-quality genome sequence of Pichia pastoris CBS7435. J Biotechnol.

[15] Liachko I, Youngblood RA, Tsui K, Bubb KL, Queitsch C, Raghuraman MK, Nislow C, Brewer BJ, Dunham MJ (2014). GC-rich DNA elements enable replication origin activity in the methylotrophic yeast Pichia pastoris. PLoS Genet.

[16] Liang S, Wang B, Pan L, Ye Y, He M, Han S, Zheng S, Wang X, Lin Y (2012). Comprehensive structural annotation of Pichia pastoris transcriptome and the response to various carbon sources using deep paired-end RNA sequencing. BMC Genomics.

[17] Vogl T, Thallinger GG, Zellnig G, Drew D, Cregg JM, Glieder A, Freigassner M (2014). Towards improved membrane protein production in Pichia pastoris: General and specific transcriptional response to membrane protein overexpression. New Biotechnol.

[18] Graf A, Gasser B, Dragosits M, Sauer M, Leparc GG, Tuchler T, Kreil DP, Mattanovich D (2008). Novel insights into the unfolded protein response using Pichia pastoris specific DNA microarrays. BMC Genomics.

[19] Graf A, Dragosits M, Gasser B, Mattanovich D (2009). Yeast systems biotechnology for the production of heterologous proteins. FEMS Yeast Res.

[20] Dikicioglu D, Wood V, Rutherford KM, McDowall MD, Oliver SG (2014). Improving functional annotation for industrial microbes: a case study with Pichia pastoris. Trends Biotechnol.

[21] Hesketh AR, Castrillo JI, Sawyer T, Archer DB, Oliver SG. Investigating the physiological response of Pichia (Komagataella) pastoris GS115 to the heterologous expression of misfolded proteins using chemostat cultures. Appl Microbiol Biotechnol. 2013. Epub ahead of print. 10.1007/s00253-013-5186-1 PMC3825213 24022610

[22] Haas BJ, Zeng Q, Pearson MD, Cuomo CA, Wortman JR (2011). Approaches to fungal genome annotation. Mycology.

[23] Ohi H, Okazaki N, Uno S, Miura M, Hiramatsu R (1998). Chromosomal DNA patterns and gene stability of Pichia pastoris. Yeast.

[24] Merchant S, Wood DE, Salzberg S (2014). Unexpected cross-species contamination in genome sequencing projects. Peer J.

[25] Bauer FF, Govender P, Bester MC (2010). Yeast flocculation and its biotechnological relevance. Appl Microbiol Biot.

[26] Russmayer H, Buchetics M, Gruber C, Valli M, Grillitsch K, Modarres G, Guerrasio R, Klavins K, Neubauer S, Drexler H (2015). Systems-level organization of yeast methylotrophic lifestyle. BMC Biol.

[27] James TC, Usher J, Campbell S, Bond U (2008). Lager yeasts possess dynamic genomes that undergo rearrangements and gene amplification in response to stress. Curr Genet.

[28] Kupiec M (2014). Biology of telomeres: lessons from budding yeast. FEMS Microbiol Rev.

[29] Meinhardt F, Schaffrath R, Larsen M (1997). Microbial linear plasmids. Appl Microbiol Biotechnol.

[30] Prielhofer R, Cartwright SP, Graf AB, Valli M, Bill RM, Mattanovich D, Gasser B (2015). Pichia pastoris regulates its gene-specific response to different carbon sources at the transcriptional, rather than the translational, level. BMC Genomics.

[31] Gasser B, Steiger MG, Mattanovich D (2015). Methanol regulated yeast promoters: production vehicles and toolbox for synthetic biology. Microb Cell Fact.

[32] Vogl T, Sturmberger L, Kickenweiz T, Wasmayer R, Schmid C, Hatzl AM, Gerstmann MA, Pitzer J, Wagner M, Thallinger GG (2016). A toolbox of diverse promoters related to methanol utilization: functionally verified parts for heterologous pathway expression in pichia pastoris. ACS Synth Biol.

[33] Prielhofer R, Maurer M, Klein J, Wenger J, Kiziak C, Gasser B, Mattanovich D (2013). Induction without methanol: novel regulated promoters enable high-level expression in Pichia pastoris. Microb Cell Fact.

[34] Liang S, Zou C, Lin Y, Zhang X, Ye Y (2013). Identification and characterization of P GCW14: a novel, strong constitutive promoter of Pichia pastoris. Biotechnol Lett.

[35] Weinhandl K, Winkler M, Glieder A, Camattari A (2014). Carbon source dependent promoters in yeasts. Microb Cell Fact.

[36] Vogl T, Glieder A (2013). Regulation of Pichia pastoris promoters and its consequences for protein production. N Biotechnol.

[37] Cereghino JL, Cregg JM (2000). Heterologous protein expression in the methylotrophic yeast Pichia pastoris. FEMS Microbiol Rev.

[38] Tamayo P, Slonim D, Mesirov J, Zhu Q, Kitareewan S, Dmitrovsky E, Lander ES, Golub TR (1999). Interpreting patterns of gene expression with self-organizing maps: methods and application to hematopoietic differentiation. Proc Natl Acad Sci U S A.

[39] Engel SR, Balakrishnan R, Binkley G, Christie KR, Costanzo MC, Dwight SS, Fisk DG, Hirschman JE, Hitz BC, Hong EL (2010). Saccharomyces genome database provides mutant phenotype data. Nucleic Acids Res.

[40] Rebnegger C, Graf AB, Valli M, Steiger MG, Gasser B, Maurer M, Mattanovich D (2014). In Pichia pastoris, growth rate regulates protein synthesis and secretion, mating and stress response. Biotechnol J.

[41] Guerfal M, Ryckaert S, Jacobs PP, Ameloot P, Van Craenenbroeck K, Derycke R, Callewaert N (2010). The HAC1 gene from Pichia pastoris: characterization and effect of its overexpression on the production of secreted, surface displayed and membrane proteins. Microb Cell Fact.

[42] Barbie DA, Tamayo P, Boehm JS, Kim SY, Moody SE, Dunn IF, Schinzel AC, Sandy P, Meylan E, Scholl C (2009). Systematic RNA interference reveals that oncogenic KRAS-driven cancers require TBK1. Nature.

[43] Unk I, Hajdu I, Blastyak A, Haracska L (2010). Role of yeast Rad5 and its human orthologs, HLTF and SHPRH in DNA damage tolerance. DNA Repair (Amst).

[44] Rodionov DA, Mironov AA, Rakhmaninova AB, Gelfand MS (2000). Transcriptional regulation of transport and utilization systems for hexuronides, hexuronates and hexonates in gamma purple bacteria. Mol Microbiol.

[45] Stincone A, Prigione A, Cramer T, Wamelink MMC, Campbell K, Cheung E, Olin-Sandoval V, Gruning NM, Kruger A, Alam MT (2015). The return of metabolism: biochemistry and physiology of the pentose phosphate pathway. Biol Rev.

[46] Bentley DR, Balasubramanian S, Swerdlow HP, Smith GP, Milton J, Brown CG, Hall KP, Evers DJ, Barnes CL, Bignell HR (2008). Accurate whole human genome sequencing using reversible terminator chemistry. Nature.

[47] Fisher S, Barry A, Abreu J, Minie B, Nolan J, Delorey TM, Young G, Fennell TJ, Allen A, Ambrogio L (2011). A scalable, fully automated process for construction of sequence-ready human exome targeted capture libraries. Genome Biol.

[48] Chin CS, Alexander DH, Marks P, Klammer AA, Drake J, Heiner C, Clum A, Copeland A, Huddleston J, Eichler EE (2013). Nonhybrid, finished microbial genome assemblies from long-read SMRT sequencing data. Nat Methods.

[49] Li H, Durbin R (2009). Fast and accurate short read alignment with Burrows-Wheeler transform. Bioinformatics.

[50] Walker BJ, Abeel T, Shea T, Priest M, Abouelliel A, Sakthikumar S, Cuomo CA, Zeng Q, Wortman J, Young SK (2014). Pilon: an integrated tool for comprehensive microbial variant detection and genome assembly improvement. PLoS One.

[51] Hyatt D, Chen GL, Locascio PF, Land ML, Larimer FW, Hauser LJ (2010). Prodigal: prokaryotic gene recognition and translation initiation site identification. BMC Bioinformatics.

[52] Lagesen K, Hallin P, Rodland EA, Staerfeldt HH, Rognes T, Ussery DW (2007). RNAmmer: consistent and rapid annotation of ribosomal RNA genes. Nucleic Acids Res.

[53] Lowe TM, Eddy SR (1997). tRNAscan-SE: a program for improved detection of transfer RNA genes in genomic sequence. Nucleic Acids Res.

[54] Grabherr MG, Haas BJ, Yassour M, Levin JZ, Thompson DA, Amit I, Adiconis X, Fan L, Raychowdhury R, Zeng Q (2011). Full-length transcriptome assembly from RNA-Seq data without a reference genome. Nat Biotechnol.

[55] Fu L, Niu B, Zhu Z, Wu S, Li W (2012). CD-HIT: accelerated for clustering the next-generation sequencing data. Bioinformatics.

[56] Wood DE, Salzberg SL (2014). Kraken: ultrafast metagenomic sequence classification using exact alignments. Genome Biol.

[57] Van der Auwera GA, Carneiro MO, Hartl C, Poplin R, Del Angel G, Levy-Moonshine A, Jordan T, Shakir K, Roazen D, Thibault J (2013). From FastQ data to high confidence variant calls: the genome analysis toolkit best practices pipeline. Curr Protoc Bioinformatics.

