# Erratum to: Comparative genomics and transcriptomics of *Pichia pastoris*

**DOI:** 10.1186/s12864-016-3109-0

**Published:** 2016-09-28

**Authors:** Kerry R. Love, Kartik A. Shah, Charles A. Whittaker, Jie Wu, M. Catherine Bartlett, Duanduan Ma, Rachel L. Leeson, Margaret Priest, Jonathan Borowsky, Sarah K. Young, J. Christopher Love

**Affiliations:** 1 Koch Institute for Integrative Cancer Research, Massachusetts Institute of Technology, 76-253, 77 Massachusetts Avenue, Cambridge, MA 02139 USA; 2 The Barbara K. Ostrom (1978) Bioinformatics and Computing Facility in the Swanson Biotechnology Center, Massachusetts Institute of Technology, Cambridge, MA 02139 USA; 3 The Broad Institute of MIT and Harvard, Cambridge, MA 02142 USA

## Erratum

In the original publication of this article [1], the accession numbers for the genomes and transcriptome listed are incorrect.

The correct details of the NCBI accession numbers can be found below:

**Availability of data and materials**

The genomic sequencing data and assembled and annotated genomes are deposited at NCBI under bioproject accession numbers PRJNA304627 (K. pastoris), PRJNA304977 (K. phaffii wildtype), and PRJNA304976 (K. phaffii GS115). RNA-seq data are deposited at NCBI under the bioproject accession numbers PRJNA311606.

In addition to this, please find the direct links to the data below:

Transcriptome study:

http://www.ncbi.nlm.nih.gov/bioproject/PRJNA311606

http://www.ncbi.nlm.nih.gov/sra?linkname=bioproject_sra_all&from_uid=311606

Genome assemblies:

http://www.ncbi.nlm.nih.gov/bioproject/PRJNA304627

http://www.ncbi.nlm.nih.gov/bioproject/PRJNA304977

http://www.ncbi.nlm.nih.gov/bioproject/PRJNA304976
