# Supplementary material for: Comparative genomics and transcriptomics of Pichia pastoris
Source: BMC Genomics. 2016 Aug 5;17:550. doi: 10.1186/s12864-016-2876-y (PMC4974788; doi:10.1186/s12864-016-2876-y)
Supplement: Additional file 2: — Supplementary figures. Figure S1. Experimental timeline for RNA sequencing of Komagatella strains. Schematic timeline for collection of RNA samples during batch cultivation of strains in shake flasks on three different carbon sources. Three independent cultivations were sampled for each time point. Figure S2. Phylogenetic comparison of K. pastoris and K. phaffii to other related yeasts. Phylogeny was generated using a concatenated, gap-free alignment of ten orthologous proteins. Phylogenetic tree was calculated using neighbor-joining, distance-based, maximum likelihood and maximum parsimony methods; reliability was assessed using bootstrapping. Clades marked with an asterisk are supported by 100 % of bootstrap replicates in all four methods. Figure S3. Gene conservation between K. pastoris and K. phaffii. a) Histogram denoting homology at the base pair level for all 1:1 orthologous genes. b) Alignment of the PGAPDH promoter element between K. pastoris and K. phaffii. c) Alignment of the PAOX1 promoter element between K. pastoris and K. phaffii. Figure S4. Copy number determination for major chromosomes in a) K. pastoris, b) K. phaffii wild-type and c) K. phaffii GS115 strains. Figure S5. Codon usage for a) K. pastoris and b) K. phaffii as determined from all coding sequences identified in genome annotation. The relative abundance observed for each codon is represented as a percentage of total codon usage for the corresponding amino acid. Figure S6. Isoform expression in K. pastoris and K. phaffii as a function of cultivation conditions. Heat maps of gene expression (log2 fpkm) for isoforms of alternatively spliced genes that alter coding sequences in a) K. pastoris and b) K. phaffii detected in initial genome annotation. Alternatively spliced genes with sufficient homology to S. cerevisiae are named, otherwise gene identifiers from genome annotation are used. Isoform expression is shown as a function of batch growth in glycerol, glucose or methanol during a 48 h cul [file 12864_2016_2876_MOESM2_ESM.pdf]

Additional file 2: Supplementary figures for

Comparative genomics and transcriptomics of *Pichia pastoris*

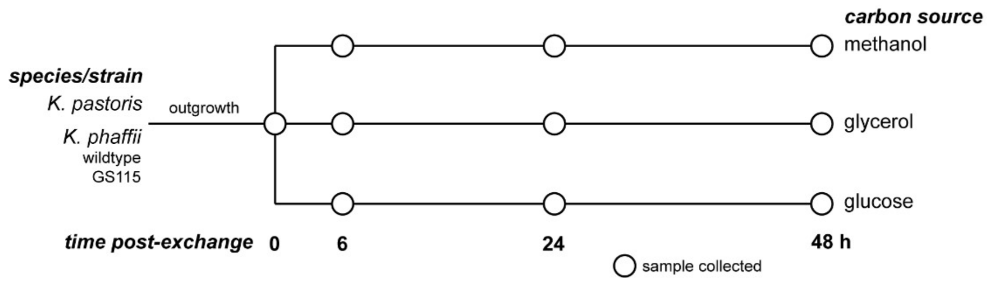

Figure S1: Experimental timeline for RNA sequencing of *Komagatella* strains.

Schematic timeline for collection of RNA samples during batch cultivation of strains in shake flasks on 3 different carbon sources. Three independent cultivations were sampled for each time point.

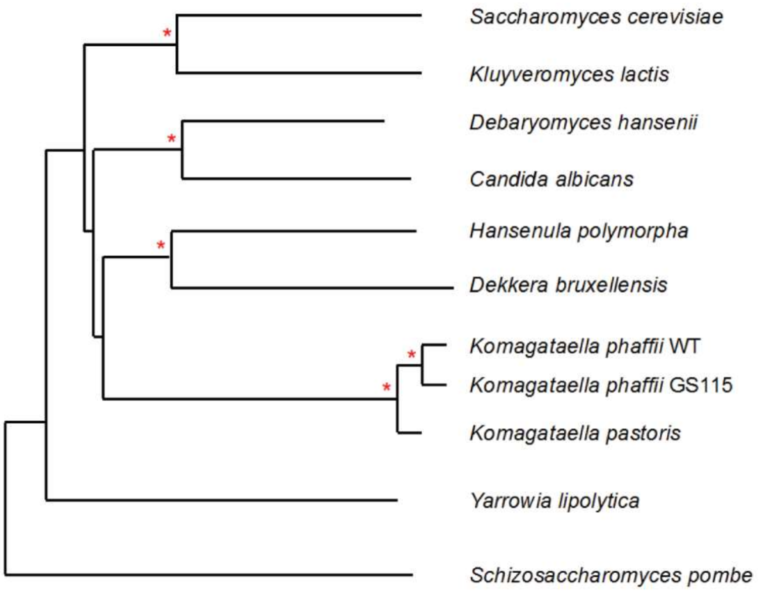

Figure S2: Phylogenetic comparison of *K. pastoris* and *K. phaffii* to other related yeasts. Phylogeny was generated using a concatenated, gap-free alignment of ten orthologous proteins. Phylogenetic tree was calculated using neighbor-joining, distance-based, maximum likelihood and maximum parsimony methods; reliability was assessed using bootstrapping. Clades marked with an asterisk are supported by 100% of bootstrap replicates in all four methods.

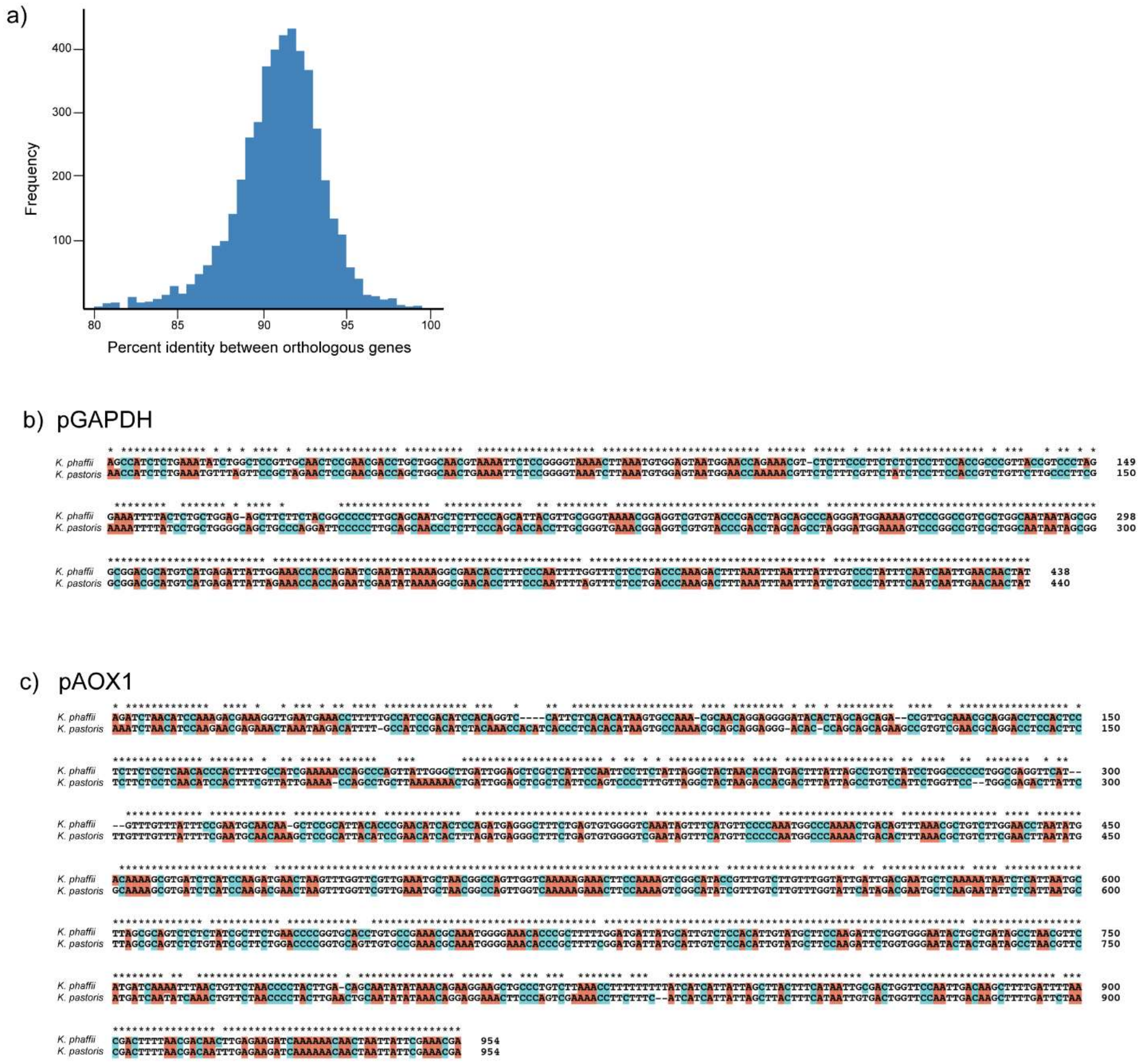

Figure S3: Gene conservation between *K. pastoris* and *K. phaffii*. a) Histogram denoting homology at the base pair level for all 1:1 orthologous genes. b) Alignment of the P_{GAPDH} promoter element between *K. pastoris* and *K. phaffii*. c) Alignment of the P_{AOX1} promoter element between *K. pastoris* and *K. phaffii*.

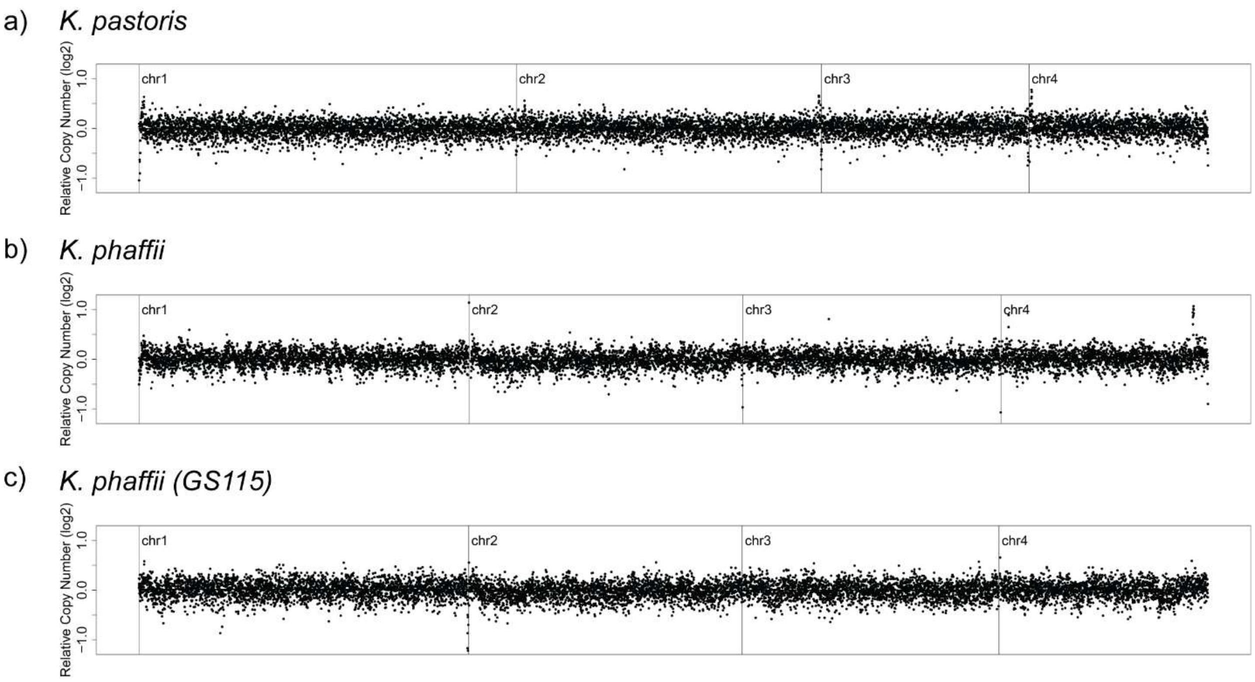

Figure S4: Copy number determination for major chromosomes in a) *K. pastoris*, b) *K. phaffii* wild-type and c) *K. phaffii* GS115 strains.

a) *K. pastoris*

First Base	Second Base: U	Second Base: C	Second Base: A	Second Base: G	Third Base
U	UUU Phe 1.14	UCU Ser 1.56	UAU Tyr 1.03	UGU Cys 1.24	U
U	UUC Phe 0.86	UCC Ser 1.04	UAC Tyr 0.97	UGC Cys 0.76	C
U	UUA Leu 1.07	UCA Ser 1.25	UAA Stop 0.00	UGA Stop 0.00	A
U	UUG Leu 1.71	UCG Ser 0.57	UAG Stop 0.00	UGG Trp 1.00	G
C	CUU Leu 1.02	CCU Pro 1.35	CAU His 1.23	CGU Arg 0.84	U
C	CUC Leu 0.51	CCC Pro 0.73	CAC His 0.77	CGC Arg 0.32	C
C	CUA Leu 0.76	CCA Pro 1.51	CAA Gln 1.20	CGA Arg 0.72	A
C	CUG Leu 0.93	CCG Pro 0.41	CAG Gln 0.80	CGG Arg 0.32	G
A	AUU Ile 1.38	ACU Thr 1.46	AAU Asn 1.07	AGU Ser 0.95	U
A	AUC Ile 0.91	ACC Thr 0.96	AAC Asn 0.93	AGC Ser 0.63	C
A	AUA Ile 0.71	ACA Thr 1.09	AAA Lys 1.04	AGA Arg 2.72	A
A	AUG Met / 1.00	ACG Thr 0.49	AAG Lys 0.96	AGG Arg 1.09	G
G	GUU Val 1.55	GCU Ala 1.62	GAU Asp 1.23	GGU Gly 1.43	U
G	GUC Val 0.87	GCC Ala 0.97	GAC Asp 0.77	GGC Gly 0.63	C
G	GUA Val 0.72	GCA Ala 1.09	GAA Glu 1.18	GGA Gly 1.45	A
G	GUG Val 0.87	GCG Ala 0.32	GAG Glu 0.82	GGG Gly 0.49	G

b) *K. phaffii*

First Base	Second Base: U	Second Base: C	Second Base: A	Second Base: G	Third Base
U	UUU Phe 1.16	UCU Ser 1.56	UAU Tyr 1.03	UGU Cys 1.24	U
U	UUC Phe 0.84	UCC Ser 1.04	UAC Tyr 0.97	UGC Cys 0.76	C
U	UUA Leu 1.07	UCA Ser 1.25	UAA Stop 0.00	UGA Stop 0.00	A
U	UUG Leu 1.74	UCG Ser 0.57	UAG Stop 0.00	UGG Trp 1.00	G
C	CUU Leu 1.03	CCU Pro 1.36	CAU His 1.23	CGU Arg 0.84	U
C	CUC Leu 0.51	CCC Pro 0.72	CAC His 0.77	CGC Arg 0.31	C
C	CUA Leu 0.74	CCA Pro 1.52	CAA Gln 1.21	CGA Arg 0.72	A
C	CUG Leu 0.91	CCG Pro 0.41	CAG Gln 0.79	CGG Arg 0.32	G
A	AUU Ile 1.40	ACU Thr 1.46	AAU Asn 1.07	AGU Ser 0.95	U
A	AUC Ile 0.89	ACC Thr 0.95	AAC Asn 0.93	AGC Ser 0.63	C
A	AUA Ile 0.71	ACA Thr 1.10	AAA Lys 1.04	AGA Arg 2.72	A
A	AUG Met / 1.00	ACG Thr 0.49	AAG Lys 0.96	AGG Arg 1.09	G
G	GUU Val 1.56	GCU Ala 1.60	GAU Asp 1.23	GGU Gly 1.43	U
G	GUC Val 0.86	GCC Ala 0.97	GAC Asp 0.77	GGC Gly 0.63	C
G	GUA Val 0.72	GCA Ala 1.10	GAA Glu 1.19	GGA Gly 1.46	A
G	GUG Val 0.87	GCG Ala 0.32	GAG Glu 0.81	GGG Gly 0.49	G

Figure S5: Codon usage for a) *K. pastoris* and b) *K. phaffii* as determined from all coding sequences identified in genome annotation. The relative abundance observed for each codon is represented as a percentage of total codon usage for the corresponding amino acid.

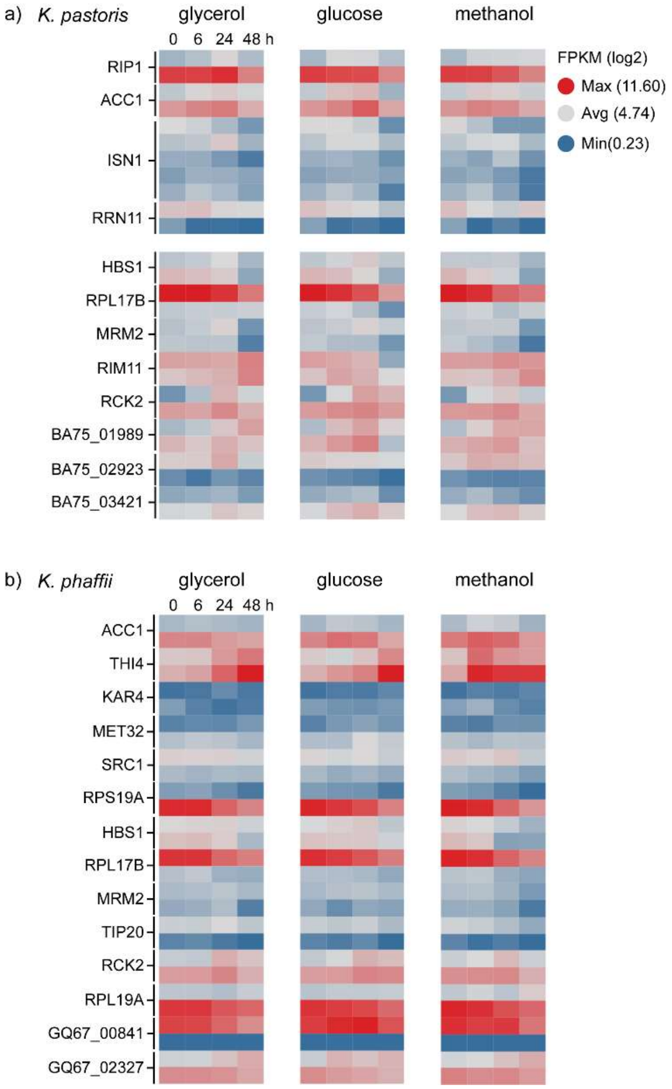

Figure S6: Isoform expression in *K. pastoris* and *K. phaffii* as a function of cultivation conditions. Heat maps of gene expression (log2 fpkm) for isoforms of alternatively spliced genes that alter coding sequences in a) *K. pastoris* and b) *K. phaffii* detected in initial genome annotation. Alternatively spliced genes with sufficient homology to *S. cerevisiae* are named, otherwise gene identifiers from genome annotation are used. Isoform expression is shown as a function of batch growth in glycerol, glucose or methanol during a 48 h cultivation period.

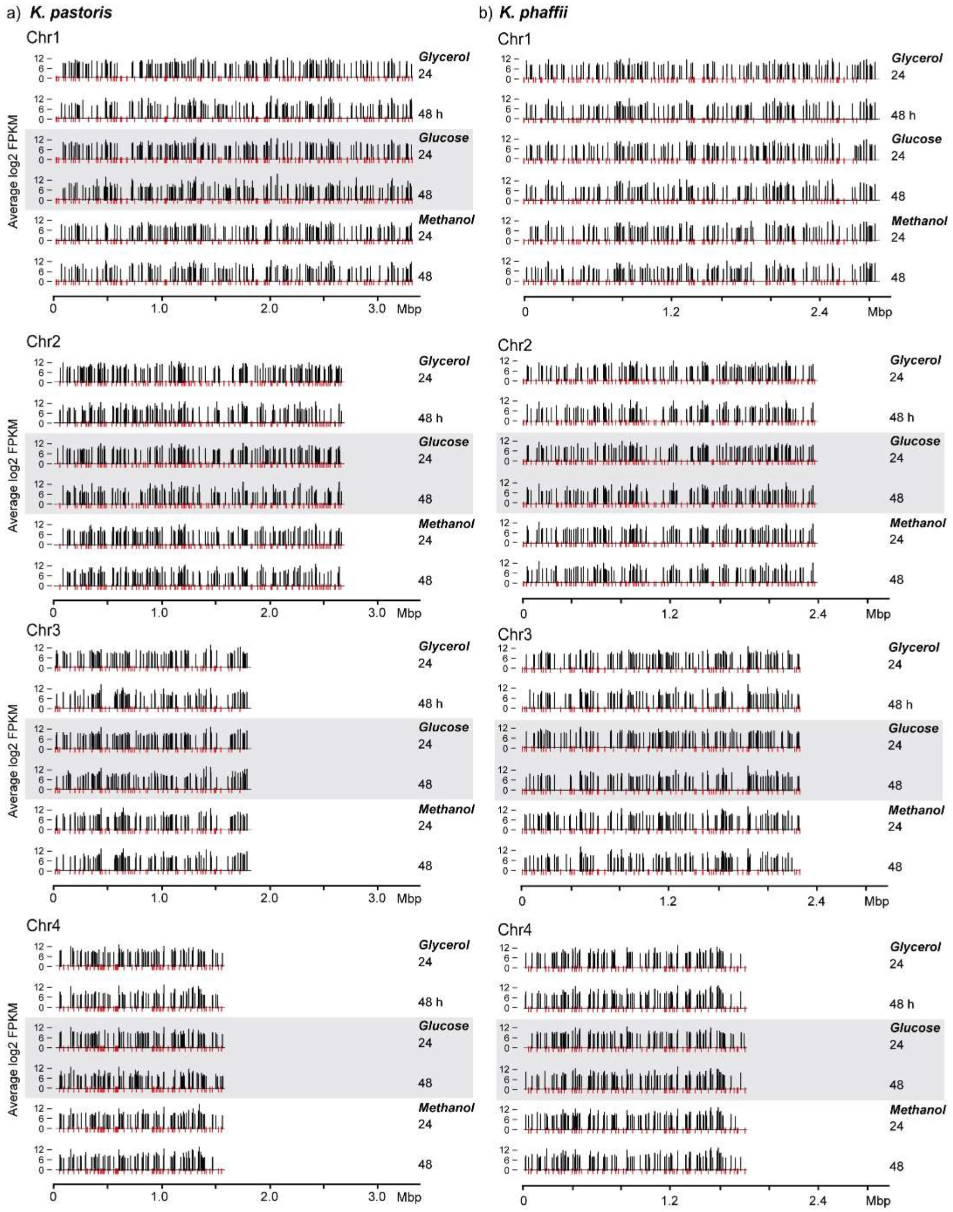

Figure S7: Chromosomal locations of highly expressed genes. Map of chromosomal location (base pair identity) for the most highly expressed genes (top 10% expression) in a) *K. pastoris* and b) *K. phaffii*. Black lines indicate gene expression level at 24 h time points during batch cultivation in either glycerol, glucose or methanol. Red lines indicate locations of GC-rich autonomously replicating sequence (GC-ARS) motifs identified by BLAST.

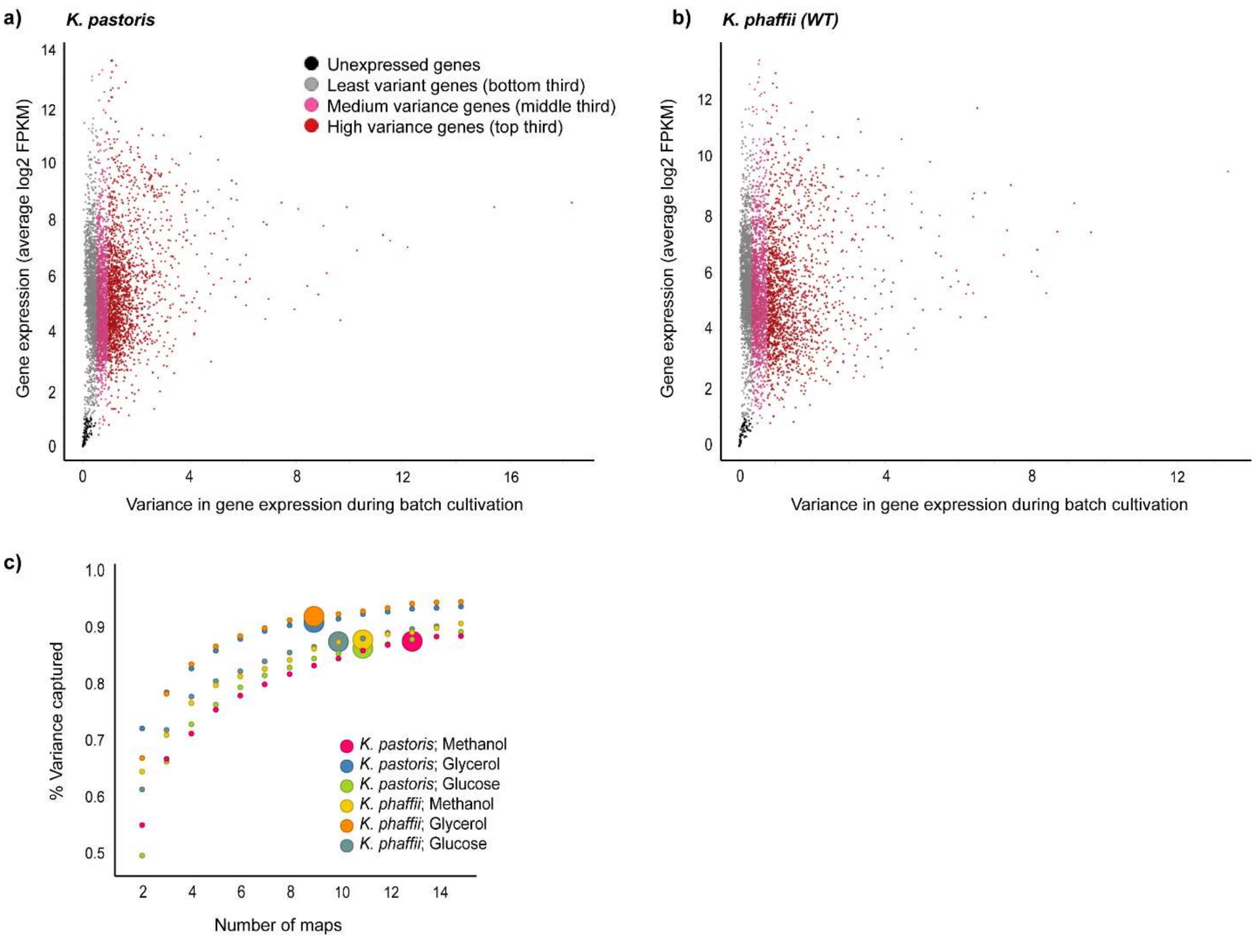

Figure S8: Variance in gene expression during batch cultivation of *K. pastoris* and *K. phaffii*. Scatter plots of the average variance versus expression observed for all annotated genes across the 10 conditional averages generated from either a) *K. pastoris* or b) *K. phaffii* expression data. Genes with average log2 fpkm <1 and variance < 0.05 were excluded from further analyses. c) Elbow analysis of input cluster number to identify optimal expression data clustering by self- organizing maps (SOMs). Large circles denote the number of clusters for each expression data set where the additional variance captured by further clustering was < 1%.

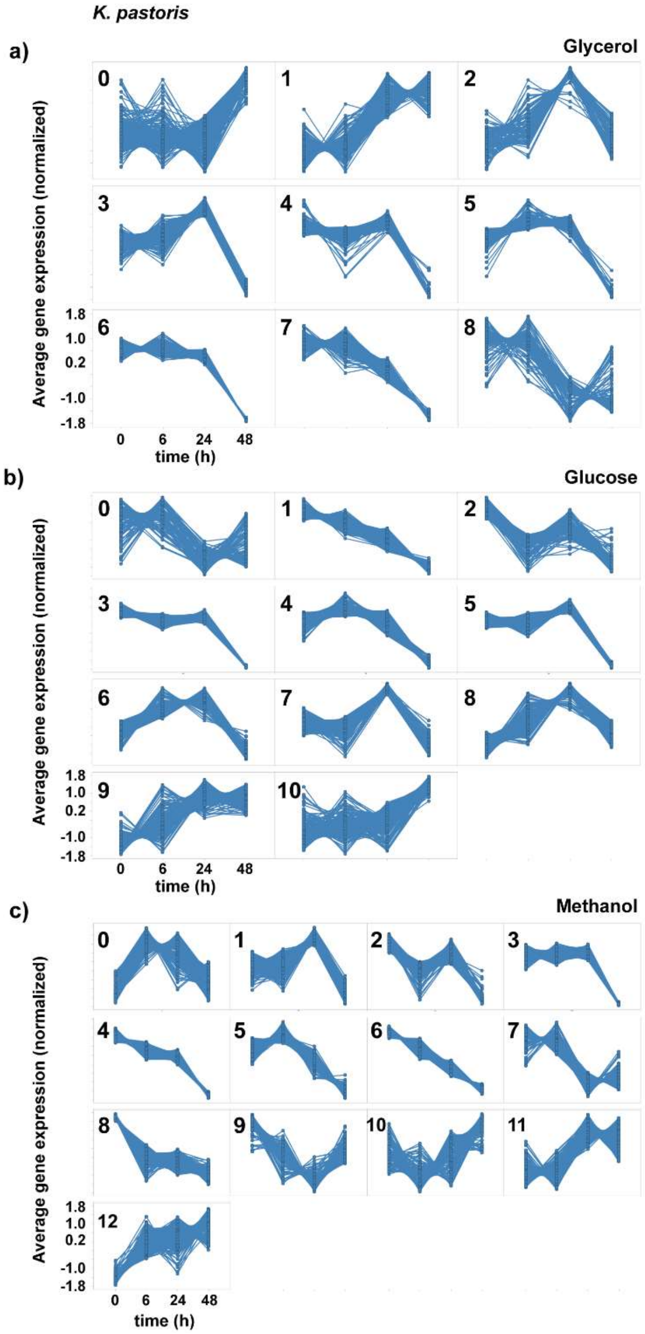

Figure S9: Gene expression phenotypes in *K. pastoris* as a function of cultivation conditions. Self-organizing maps (SOMs) of genes changing expression similarly in *K. pastoris* during a 48 h batch cultivation in a) glycerol, b) glucose or c) methanol.

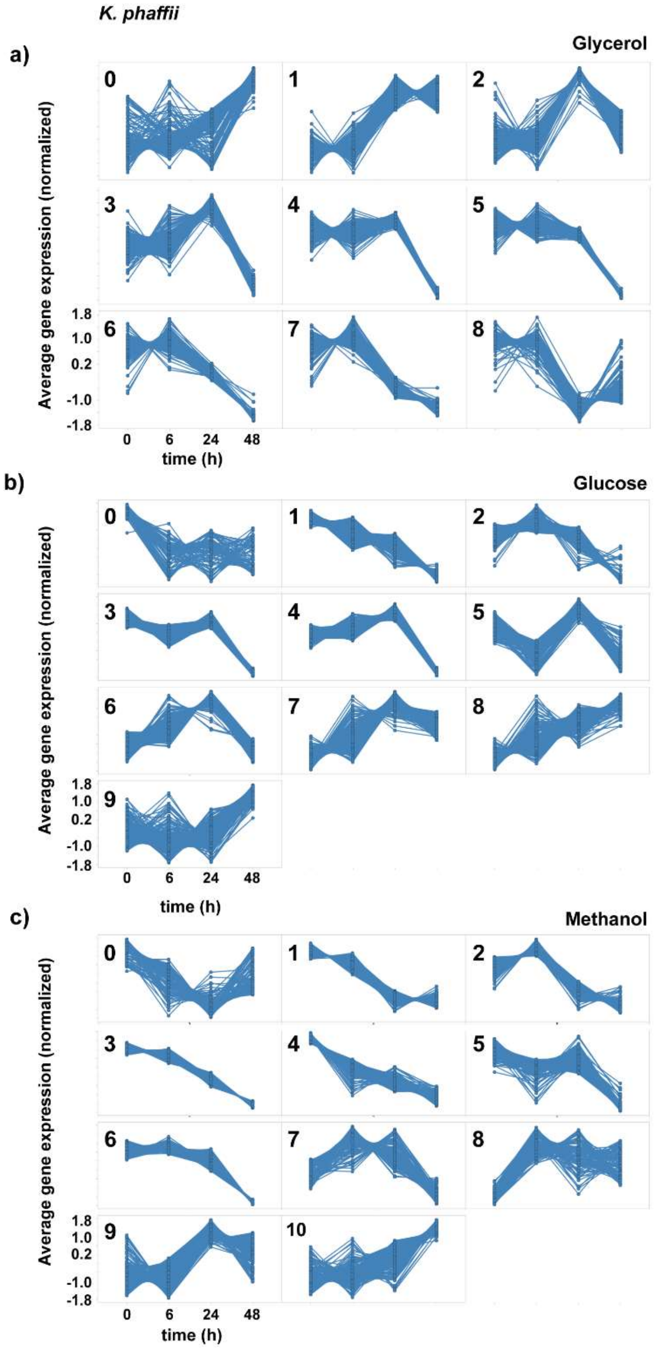

Figure S10: Gene expression phenotypes in *K. phaffii* as a function of cultivation conditions. Self-organizing maps (SOMs) of genes changing expression similarly in *K. phaffii* during a 48 h batch cultivation in a) glycerol, b) glucose or c) methanol.

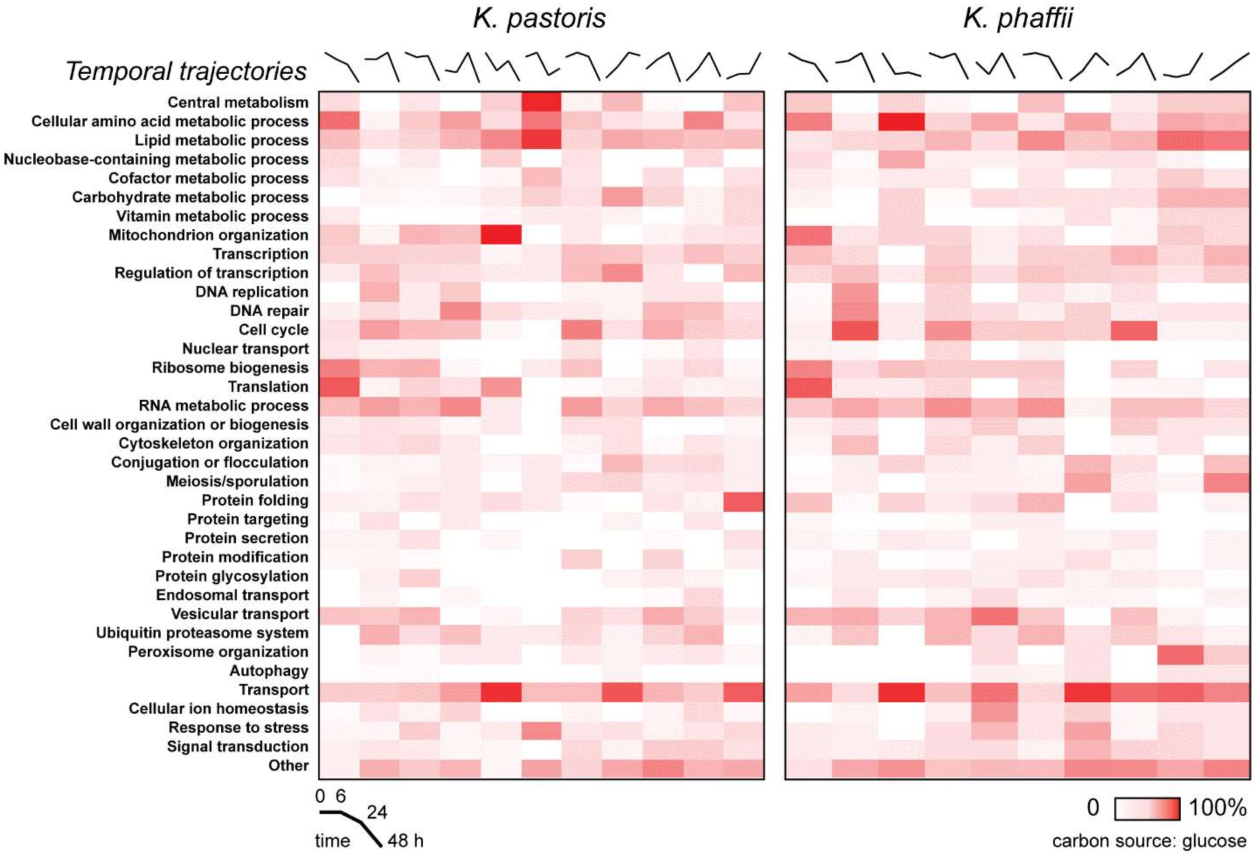

Figure S11: Biological process enrichment as a function of cultivation in glucose. Heat map representation of the enrichment of GO biological process terms for expression phenotypes observed in *K. pastoris* and *K. phaffii* during a 48 h batch cultivation in glucose as characterized by self-organizing maps (SOMs). Representative temporal trajectories of gene expression were generated for each SOM by averaging expression data at each time point for genes present within a given map. Color density relates to the number of genes assigned to a particular process as a percentage of the total number genes present in a particular expression phenotype or map.

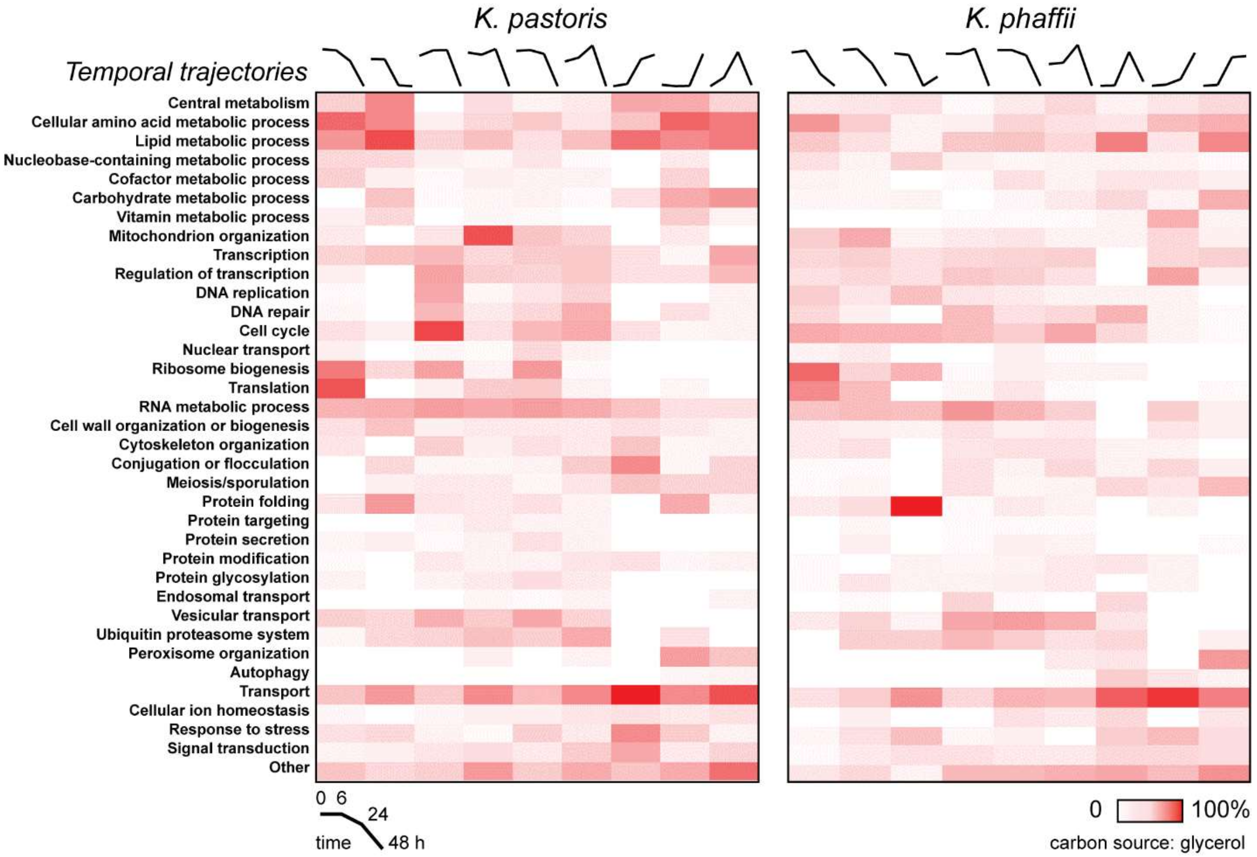

Figure S12: Biological process enrichment as a function of cultivation in glycerol. Heat map representation of the enrichment of GO biological process terms for expression phenotypes observed in *K. pastoris* and *K. phaffii* during a 48 h batch cultivation in glycerol as characterized by self-organizing maps (SOMs). Representative temporal trajectories of gene expression were generated for each SOM by averaging expression data at each time point for genes present within a given map. Color density relates to the number of genes assigned to a particular process as a percentage of the total number genes present in a particular expression phenotype or map.

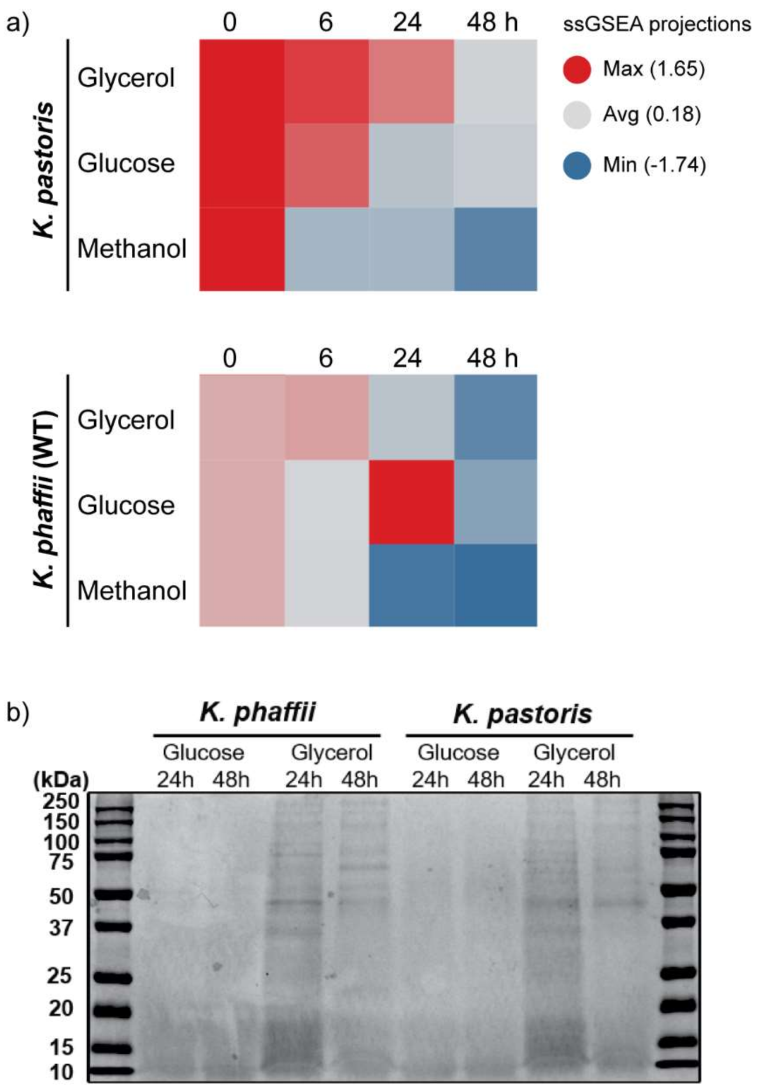

Figure S13: Secretory pathway protein expression in *K. pastoris* and *K. phaffii*. a) Row normalized single set Gene Set Enrichment Analysis (ssGSEA) projections for 170 proteins bearing a signal peptide as identified by Signalp. b) SDS-PAGE analysis of host-cell protein expression in supernantants during batch cultivation of *K. pastoris* and *K. phaffii* for 48h in glucose or glycerol-containing media.

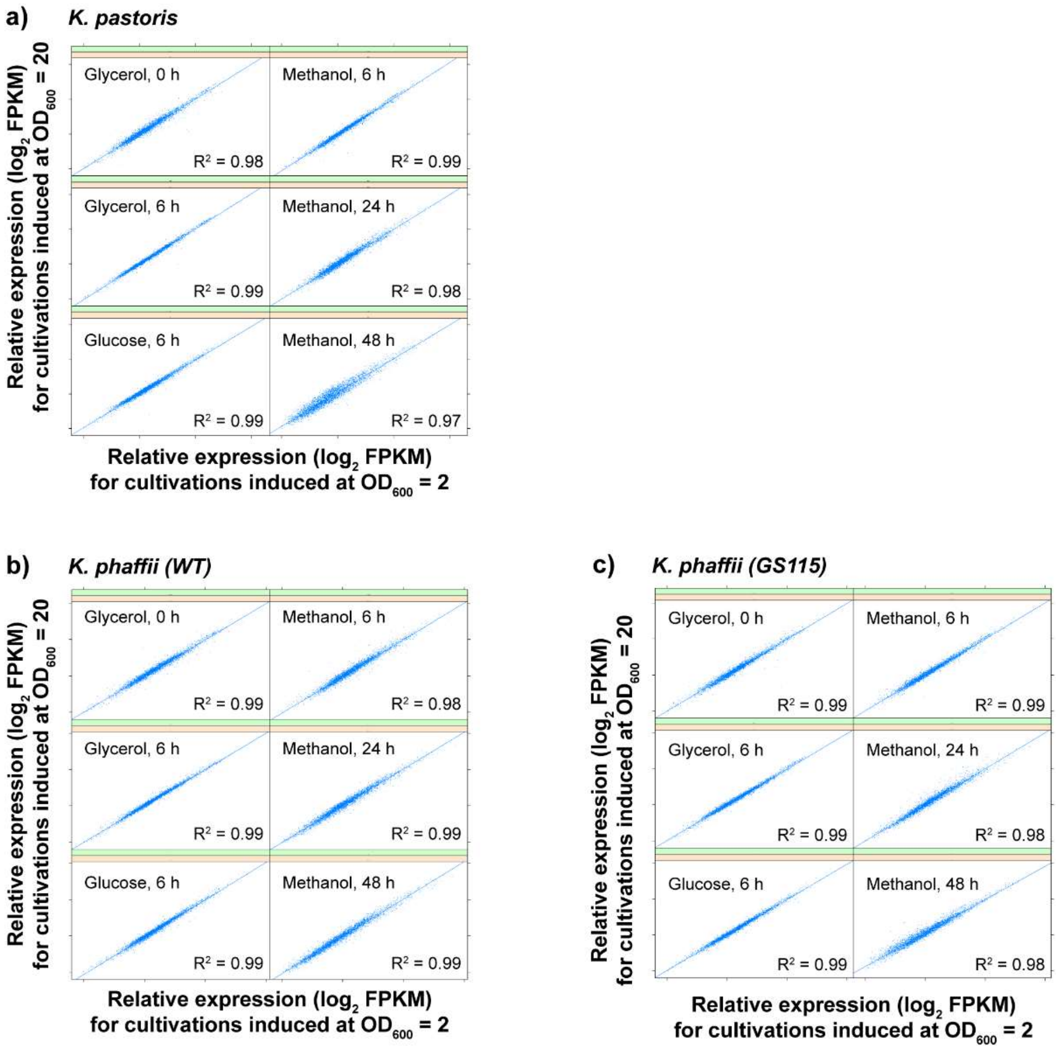

Figure S14: Correlation of gene expression at two different cultivation densities.

Scatter plots of gene expression between similar cultivation conditions for a) *K. pastoris*, b) wildtype *K. phaffii*, and c) *K. phaffii* GS115 grown at two different cell densities. Density A corresponds to cultures outgrown to OD_{600} = 2.0 prior to sampling and Density B corresponds to cultures outgrown to OD_{600} = 20 prior to sampling. Pearson correlation coefficients were calculated from expression vectors that were averages of three biological replicates for each cultivation condition and density.
