# Supplementary material for: Comparative genomics and transcriptomics of Pichia pastoris
Source: BMC Genomics. 2016 Aug 5;17:550. doi: 10.1186/s12864-016-2876-y (PMC4974788; doi:10.1186/s12864-016-2876-y)
Supplement: Additional file 9: Table S8. — GO annotation of genes that are highly and differentially expressed in a particular carbon source during fermentation. Genes classified here were highly (top 10 % expression) and differentially expressed (log2-fold change >2, p < 0.05) during fermentation on the carbon source listed. (PDF 393 kb) [file 12864_2016_2876_MOESM9_ESM.pdf]

Table S8: GO annotation of genes that are highly and differentially expressed in a particular carbon source during fermentation. Genes classified here were highly (top 10% expression) and differentially expressed (log2-fold change > 2, p < 0.05) during fermentation on the carbon source listed.

Methanol-specific genes

GO biological process category	Number of genes
unassigned (no gene name)	80
transport	16
cellular amino acid metabolic process	12
response to stress	10
other	8
lipid metabolic process	8
protein folding	7
carbohydrate metabolic process	7
central metabolism	6
unknown	5
peroxisome organization	5
meiosis/sporulation	4
regulation of transcription	4
signal transduction	3
cofactor metabolic process	2
mitochondrion organization	2
cell wall organization or biogenesis	2
transcription	2
autophagy	2
ubiquitin proteasome system	2
protein secretion	1
translation	1
cytoskeleton organization	1

Glycerol-specific genes

GO biological process category	Number of genes
unassigned	24
transport	10
lipid metabolic process	8
central metabolism	5
cellular amino acid metabolic process	4
peroxisome organization	3
other	3
response to stress	3
cell wall organization or biogenesis	2
carbohydrate metabolic process	2
unknown	2
DNA replication	1
ubiquitin proteasome system	1
cofactor metabolic process	1
protein glycosylation	1
DNA repair	1
regulation of transcription	1
meiosis/sporulation	1
mitochondrion organization	1
**Total genes**	**74**

Glucose-specific genes

Row Labels	Number of genes
unassigned	17
transport	4
central metabolism	4
lipid metabolic process	4
cellular amino acid metabolic process	3
cell wall organization or biogenesis	2
cofactor metabolic process	2
response to stress	1
unknown	1
RNA metabolic process	1
DNA replication	1
carbohydrate metabolic process	1
regulation of transcription	1
protein glycosylation	1
Protein folding	1
**Total genes**	**44**

protein modification	1		
DNA repair	1		
RNA metabolic process	1		
cellular ion homeostasis	1		
nucleobase-containing metabolic process	1		
vesicular transport	1		
vitamin metabolic process	1		
conjugation or flocculation	1		
ribosome biogenesis	1		
**Total genes**	**199**		
