# Supplementary material for: Comparative genomics and transcriptomics of Pichia pastoris
Source: BMC Genomics. 2016 Aug 5;17:550. doi: 10.1186/s12864-016-2876-y (PMC4974788; doi:10.1186/s12864-016-2876-y)
Supplement: Additional file 11: Table S10. — List of mutational variants in GS115 and their potential to affect protein function. Lower SIFT scores indicate higher degrees of conservation for the native amino acid affected by the nucleotide change. Scores below the default threshold of 0.05 are highly likely to affect protein function. The differential expression summary column consists of three numbers in x/y/z format, where x corresponds to the number of culture conditions (out of 10 total) where the gene is found to be significantly differentially expressed between GS115 and wildtype K. phaffii (log fold change >2, p <0.05), y is the number of times the expression comparison tests are significantly higher in wildtype K. phaffii, and z is the number of times the expression comparison test are significantly higher in GS115. (PDF 339 kb) [file 12864_2016_2876_MOESM11_ESM.pdf]

Table S10: List of mutational variants in GS115 and their potential to affect protein function. Lower SIFT scores indicate a higher potential for the amino acid affected by the nucleotide change to impact protein function. Scores below the default threshold of 0.05 are highly likely to affect protein function. The differential expression summary column consists of three numbers in x/y/z format, where x corresponds to the number of culture conditions (out of 10 total) where the gene is found to be significantly differentially expressed between GS115 and wildtype *K. phaffii* (log fold change > 2, p < 0.05), y is the number of times the expression comparison tests are significantly higher in wildtype *K. phaffii*, and z is the number of times the expression comparison test are significantly higher in GS115.

Gene name	Chromosome	Position	Nucleotide change	Amino acid change	Domain impacted	SIFT score	Differential expression summary
MTG1	chr2	1928634	Gtg/Atg	V193M	FeoB_N	0.02	0/0/0
YDR131C	chr2	1792171	aGt/aAt	S469N	Outside domain	0.51	0/0/0
LOS1	chr2	1158548	Gtg/Atg	V56M	Xpo1	0.14	0/0/0
YH93_SCHPO	chr2	637700	Gta/Ata	V298I	DUF3844	N/O	0/0/0
DOP1	chr2	342975	Ttt/Gtt	F692V	Outside domain	0.14	0/0/0
VPS35	chr1	2322612	gCt/gTt	A681V	Outside domain	0.11	0/0/0
SMI1*	chr1	2282386	Gtc/Atc	V157I	SML1_KNR4	0.24	0/0/0
MSS4	chr1	1399834	aCa/aTa	T240I	Outside domain	0.01	0/0/0
YMR087W	chr1	942034	3'UTR	N/A		N/A	0/0/0
CWH43	chr1	786743	aGt/aAt	S870N	Outside domain	0.16	0/0/0
TGL1#	chr1	677459	Gac/Aac	D154N	Abhydrolase_1	0	0/0/0
RAD5	chr3	696403	tCc/tTc	S752F	Outside domain	0.02	0/0/0
DNL4	chr3	1411937	atG/atA	M285I	DNA_ligase_A_M	0.18	0/0/0
RPO31	chr4	637369	gAa/gGa	E779G	RPOLA_N	0.03[§]	0/0/0
YIR007W	chr4	685226	gGt/gAt	G324D	Outside domain	0	0/0/0

CRH1	chr4	1129990	Gtt/Att	V9I	Blyco_hydro_16	N/O	0/0/0
COS111	chr4	1588323	aGt/aAt	S235N	Outside domain	0.48	0/0/0
MRPS28	chr2	714857	gGc/gAc	G256D	Outside domain	1	1/1/0
UTP10	chr2	2300595	gCc/gTc	A1112V	Outside domain	0.2	1/1/0
YMR253C	chr2	1066732	gCt/gTt	A320V	EamA	0.01	1/1/0
DBP8#	chr2	709207	Gac/Aac	D269N	SRP54N	0.02§	1/0/1
HIS4	chr1	1703668	Tgc/Cgc	C557R	Histidinol_dh	0	1/0/1
FMS1	chr3	1810776	tgG/tgA	W57*	NAD_binding_8	N/A-stop	1/0/1
NAB3	chr3	414260	Gag/Aag	E227K	Outside domain	0.03	1/0/1
LCB5	chr3	794870	Cct/Tct	P259S	Outside domain	0.43	1/0/1
PEX28	chr3	1722712	Gtg/Atg	V448M	Pex24p	0	1/0/1
PRP21#	chr4	217542	Gtt/Att	V107I	Outside domain	0.27	1/0/1
MASY_PICAN	chr4	425779	Cct/Tct	P478S	Malate_synthase	0§	1/0/1
AVT2	chr4	678710	Gag/Aag	E359K	Aa_trans	0.33	1/0/1
SFB3	chr4	988521	Gct/Act	A3T	Outside domain	0§	1/0/1
PIR_HUMAN	chr1	1675511	Taa/Caa	*304Q	Outside domain	N/A-stop	2/2/0
PEX22_PICPA	chr3	1131355	Ccc/Tcc	P34S	Outside domain	0.73	2/2/0
SMC3	chr2	1805135	Cac/Tac	H2Y	Outside domain	1	3/1/2
EXO1	chr4	989019	Cca/Tca	P83S	XPGN	0.15	3/1/2
YDR248C	chr1	1973428	cCg/cTg	P9L	Outside domain	0.13	6/6/0

*Denotes a stop codon in translated gene product

# Denotes genes with a controversial annotation

§ Denotes low confidence SIFT prediction.

N/A denotes that SIFT analysis is not applicable based on the mutation type.

N/O denotes that no orthogroups were found for this gene in the Fungal Orthogroups Repository.
