# Supplementary material for: Comparative genomics and transcriptomics of Pichia pastoris
Source: BMC Genomics. 2016 Aug 5;17:550. doi: 10.1186/s12864-016-2876-y (PMC4974788; doi:10.1186/s12864-016-2876-y)
Supplement: Additional file 12: Table S11. — Summary of mutational variants found in GS115. (PDF 176 kb) [file 12864_2016_2876_MOESM12_ESM.pdf]

Table S11: Summary of genic mutational variants found in GS115

	GS115 to WT	WT to GS115	Reciprocal mutations with impact on protein sequence
Insertions	5	4	0
Deletions	25	18	0
Synonymous coding	23	43	N/A
Non-synonymous coding	38	63	32
Start gained	1	0	0
Start lost	1	0	0
Stop gained	1	1	1
Stop lost	1	1	1
5' UTR	2	3	0
3' UTR	7	11	1
